# Autophagy activation by urolithin-a derivative UA-36 mitigates Friedreich’s ataxia pathologies induced by frataxin deficiency

**DOI:** 10.1186/s43556-026-00457-w

**Published:** 2026-06-04

**Authors:** Qichao Gong, Tiansu Liu, Xiao Han, Ruiming Zhang, Xinlei Liu, Bocheng Xiong, Tahir Ali, Jianxiang Huang, Yongmei Xie, Shupeng Li, Xifei Yang

**Affiliations:** 1 State Key Laboratory of Chemical Oncogenomics, School of Chemical Biology and Biotechnology, Peking University Shenzhen Graduate School, Shenzhen, 518055 China; 2 Shenzhen Key Laboratory of Modern Toxicology, Shenzhen Medical Key Discipline of Health Toxicology (2020-2024), Shenzhen Center for Disease Control and Prevention, Shenzhen, 518055 China; 3 School of Public Health, Key Laboratory of Environmental Pollution Monitoring and Disease Control, Ministry of Education, Guizhou Medical University, Guiyang, 561113 China; 4 Department of Toxicology, School of Public Health, Shanxi Medical University, Taiyuan, Shanxi 030001 China; 5 State Key Laboratory of Biotherapy and Cancer Center, West China Hospital, Sichuan University, and Collaborative Innovation Center of Biotherapy, Chengdu, 610041 PR China; 6 Functional Microbiology Research and Development Center, Research Institute of Tsinghua University in Shenzhen, Shenzhen, Guangdong 518055 China; 7 Institute for Future Human Habitats, Tsinghua University Shenzhen International Graduate School, Shenzhen, Guangdong Province China

**Keywords:** Friedreich’s ataxia, Autophagy, UA-36, Frataxin, Mitochondrial dysfunction

## Abstract

Friedreich’s ataxia (FA) is a progressive autosomal recessive neurodegenerative disorder caused by frataxin (*FXN*) deficiency, resulting in mitochondrial dysfunction, oxidative stress, defective autophagy, and progressive motor impairment. Despite extensive efforts, effective disease-modifying therapies for FA remain lacking. Here, we investigate the therapeutic efficacy and underlying mechanisms of UA-36, a novel water-soluble and bioavailable derivative of urolithin A, in cellular and animal models of FA. In *Fxn*-knockdown N2a cells, UA-36 significantly restored FXN protein levels, enhanced autophagic flux, improved mitochondrial function, and attenuated oxidative stress–induced damage. In vivo, oral administration of UA-36 for eight weeks in YG8R transgenic mice, a well-established FA model, markedly improved motor coordination, gait performance, and skeletal muscle strength. Histological and ultrastructural analyses revealed substantial protection against cerebellar Purkinje cell loss and iron deposition, cardiac hypertrophy, and the degree of skeletal muscle atrophy and fibrosis. Proteomic analysis of cerebellar tissue demonstrated that UA-36 robustly reprograms the FA-associated molecular landscape by upregulating pathways related to autophagy, mitochondrial biogenesis, oxidative phosphorylation, and redox homeostasis, while suppressing apoptosis and neuroinflammatory signaling. Together, these findings identify UA-36 as a promising lead compound and provide compelling evidence that therapeutic enhancement of autophagy and mitochondrial quality control represents a viable, mechanism-based strategy for the treatment of FA.

**Supplementary Information:**

The online version contains supplementary material available at 10.1186/s43556-026-00457-w.

## Introduction

Friedreich’s ataxia (FA) is a devastating autosomal recessive neurodegenerative disorder primarily caused by guanine-adenine-adenine (GAA) triplet expansions in the frataxin *(FXN)* gene, leading to a critical deficiency in the mitochondrial protein frataxin [1–3]. This deficiency disrupts iron-sulfur cluster biogenesis, resulting in pervasive mitochondrial dysfunction, iron accumulation, and elevated oxidative stress [4]. These core pathologies drive the progressive neurodegeneration of the dorsal root ganglia, spinocerebellar tracts, and cerebellum, manifesting clinically as ataxia, muscle weakness, and cardiomyopathy. While Omaveloxolone has recently been approved as the first therapy for FA, its long-term safety profile remains a concern, and it does not fully halt disease progression [5, 6]. Therefore, the exploration of novel treatment options that target the underlying molecular mechanisms remains an urgent clinical priority [7].

A central consequence of frataxin deficiency is the widespread impairment of cellular quality control. Within this pathological cascade, the role of autophagy, a crucial degradative pathway for maintaining homeostasis, is complex and critically important [4, 8]. Evidence across models presents a paradox: studies in Drosophila and mice indicate that frataxin loss enhances the initiation of mitophagy but also leads to an accumulation of autophagic substrates, such as p62, suggesting a concurrent impairment in autophagic flux that prevents completion of the process [9, 10]. This blockade results in the accumulation of dysfunctional organelles, exacerbating oxidative stress and neurodegeneration. Furthermore, emerging research reveals that the protective effects of modulating mitochondrial proteins, such as mitofusin (Marf), in FA models are linked to alleviating ER stress [11], highlighting an underappreciated autophagy-endoplasmic reticulum (ER) stress axis in disease progression. Therefore, while the precise mechanism remains incompletely defined, therapeutically restoring autophagic flux represents a promising strategy.

Natural compounds that modulate autophagy offer compelling therapeutic leads [12]. Urolithin A (UA), a gut-microbiome-derived metabolite of ellagitannins, is a potent inducer of autophagy and mitophagy [13]. Its ability to improve mitochondrial health and clear cellular debris has been shown to benefit various age-related and neurodegenerative conditions [14]. However, its clinical translation is often limited by suboptimal pharmacokinetics [15].

In this study, we developed UA-36, a novel urolithin A derivative with improved water solubility and bioavailability, designed to overcome these pharmacokinetic limitations and specifically target FA-associated pathologies. We hypothesized that UA-36 would restore impaired autophagic flux in FXN-deficient states, thereby rescuing mitochondrial integrity and attenuating neurodegeneration. Using a combination of *Fxn*-knockdown cells and the YG8R transgenic mouse model, we demonstrate that UA-36 effectively restores frataxin protein levels, activates autophagic flux, rescues mitochondrial function, and ameliorates behavioral and histopathological deficits. Our work identifies UA-36 as a promising lead compound and provides compelling evidence that targeted autophagy activation is a viable therapeutic strategy for Friedreich’s ataxia.

## Results

### UA-36 restores autophagic flux and mitochondrial integrity in FXN-deficient neuronal cells

**Fig. 1:**
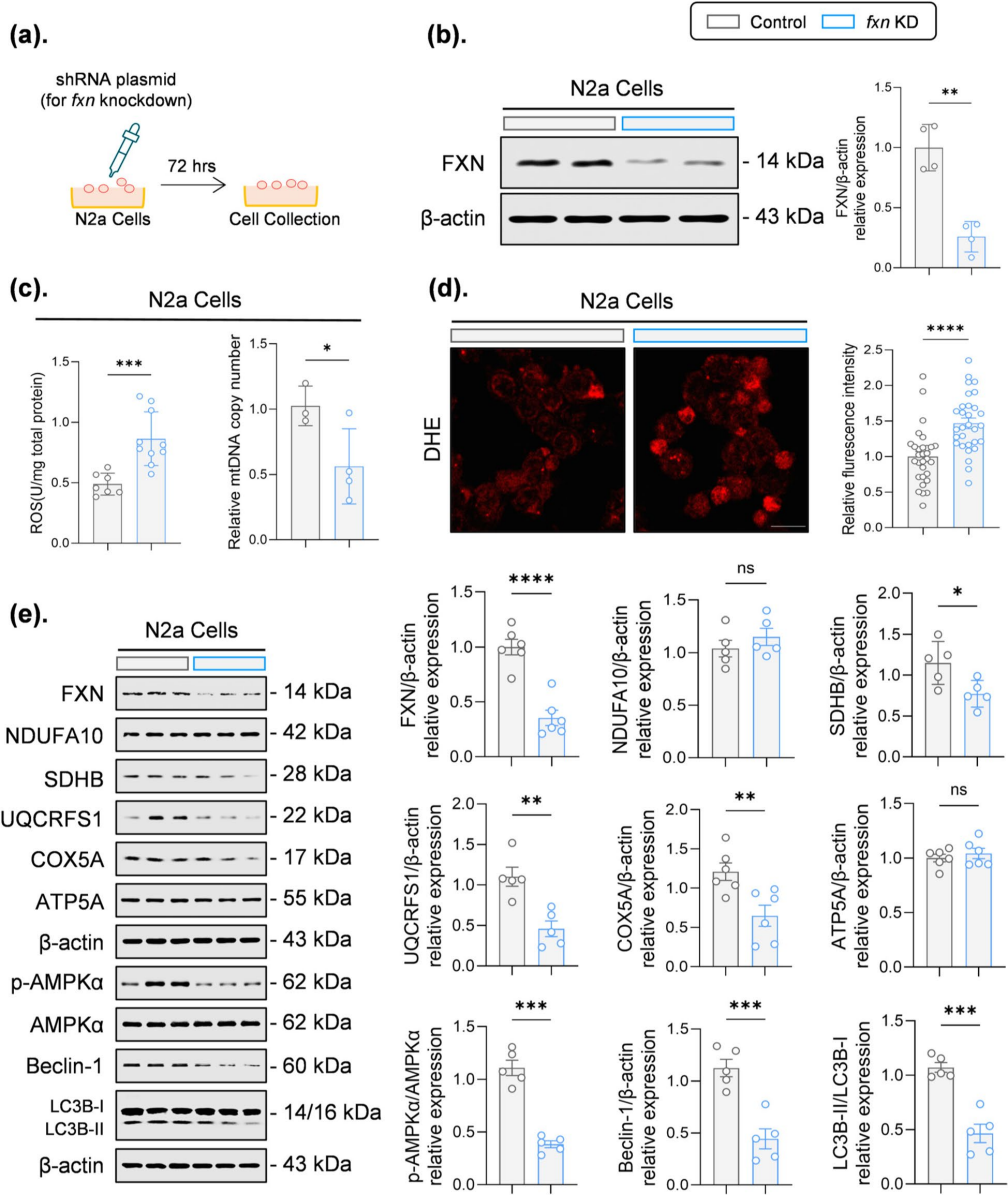
FXN knockdown induces mitochondrial dysfunction and autophagy impairment in N2a cells (**a**) Schematic diagram illustrating the experimental strategy for selective silencing of FXN in N2a cells using shRNA-mediated knockdown. Cells were transfected with control or FXN-targeting shRNA plasmid and collected after 72 h for subsequent analyses (**b**) Representative immunoblots (left) and quantitative analysis (right) confirming robust reduction of FXN protein levels in FXN-knockdown (FXN KD) cells compared with control cells. β-actin served as a loading control (*n* = 4 independent experiments) (**c**) FXN deficiency significantly increased intracellular reactive oxygen species (ROS) levels (left) and reduced relative mitochondrial DNA (mtDNA) copy number (right), indicating impaired mitochondrial function and elevated oxidative stress. mtDNA copy number was determined by qPCR quantification of mitochondrial versus nuclear DNA (*n* = 4–10 independent experiments) (**d**) Representative dihydroethidium (DHE) staining images (left) and quantification of fluorescence intensity (right) demonstrating elevated oxidative stress in FXN-knockdown cells. Increased DHE fluorescence indicates enhanced superoxide production. Scale bar = 30 μm (*n *= 30 cells from three independent experiments) (**e**) Representative immunoblots (left) and quantitative analysis (right) of FXN, NDUFA10, SDHB, UQCRFS1, COX5A, ATP5A, autophagy-related proteins (p-AMPKα, Beclin-1, LC3B-II). FXN deficiency markedly reduced mitochondrial complex protein levels, suppressed AMPK phosphorylation, and dysregulated autophagic markers (*n* = 5–6 independent experiments). All quantitative data are presented as mean ± SEM for *n* ≥ 4, or mean ± SD for *n* < 4. Statistical significance was determined by an unpaired two-tailed Student’s t-test. *p < 0.05, **p < 0.01, ***p < 0.001, ****p < 0.0001; ns, not significant. FXN, frataxin; KD, knockdown; ROS, reactive oxygen species; mtDNA, mitochondrial DNA; DHE, dihydroethidium; ETC, electron transport chain

To model the cellular pathology of FA and evaluate whether the novel compound UA-36 can rescue frataxin (FXN) deficiency-induced dysfunction, we established a cellular model using *fxn*-knockdown (*fxn*-KD) N2a neuronal cells via short hairpin RNA (shRNA) (Fig. 1a, b). Initially, the cytotoxicity of UA-36 was first assessed across a range of concentrations (1–50 µM), showing no significant reduction in cell viability below 20 µM (Fig. S1a). FXN loss triggered severe mitochondrial dysfunction, characterized by reduced mitochondrial DNA (mtDNA) content and elevated reactive oxygen species (ROS), as evidenced by dihydroethidium (DHE) staining and complementary assays (Fig. 1c, d). Molecular profiling further revealed an imbalance in mitochondrial dynamics and biogenesis, with upregulated levels of the fission protein dynamin-related protein 1 (DRP1) and downregulation of key markers for fusion (mitofusin 1, MFN1), biogenesis (peroxisome proliferator-activated receptor gamma coactivator 1-alpha, PGC-1α), and oxidative phosphorylation (succinate dehydrogenase complex iron sulfur subunit B, SDHB; ubiquinol-cytochrome c reductase core protein 1, UQCRFS1; cytochrome c oxidase subunit 5 A, COX5A) (Fig. 1e; Fig. S1b). However, optic atrophy 1 (OPA1), NADH: ubiquinone oxidoreductase subunit A10 (NDUFA10), and ATP synthase subunit alpha (ATP5A) remained unchanged, suggesting a selective disruption of mitochondrial integrity rather than a global loss of all respiratory machinery. In parallel, cellular antioxidant defence systems were compromised, evidenced by decreased protein levels of nuclear factor erythroid 2-related factor 2 (NRF2), heme oxygenase-1 (HO-1), and nitrogen fixation 1 homolog (NFS1), alongside diminished phosphorylation of AMP-activated protein kinase alpha (AMPKα) (Fig. 1e; Fig. S1b). Moreover, autophagic activity was also impaired, as indicated by reduced levels of Beclin-1 and the lipidated form of microtubule-associated protein 1A/1B-light chain 3 (LC3B-II) (Fig. 1e). These results indicate that the deficiency of frataxin leads to structural disorders in the mitochondrial electron transport chain and disturbances in the antioxidant system, accompanied by changes in the pathways of autophagy.

**Fig. 2:**
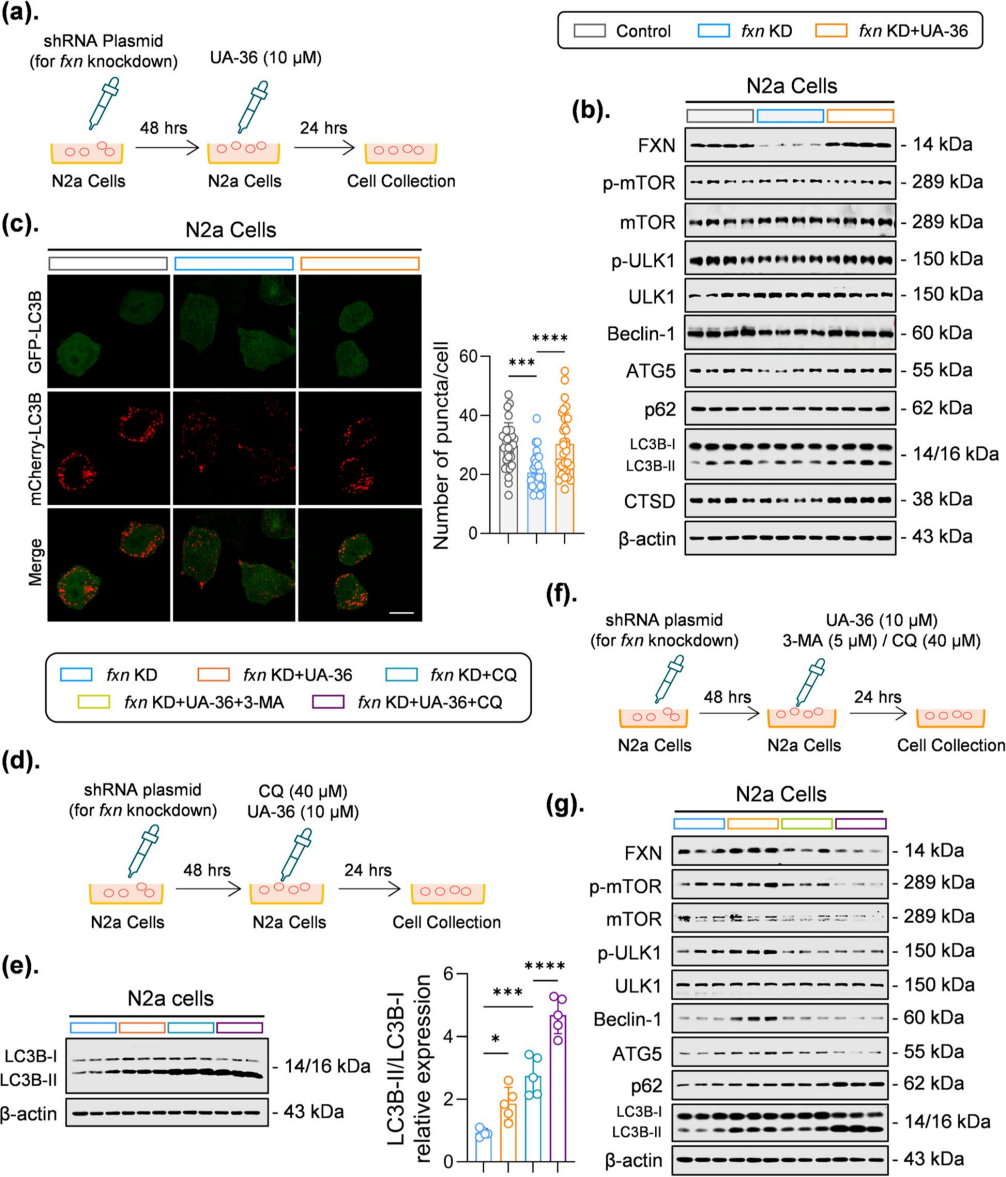
UA-36 restores FXN protein homeostasis through enhancing autophagic flux (**a**) Schematic diagram illustrating the experimental timeline for UA-36 treatment in Fxn-knockdown N2a cells. Cells were transfected with the shRNA plasmid for 48 h to knock down FXN, followed by UA-36 (10 μM) treatment for 24 h before collection (**b**) Representative immunoblots showing FXN and autophagy-related proteins in control, FXN knockdown (fxn KD), and fxn KD + UA-36-treated N2a cells. UA-36 increased FXN protein levels and modulated autophagy markers, including phosphorylation of ULK1 (activating), Beclin-1, ATG5, LC3B-II (lipidation), and CTSD (maturation), while total mTOR and p62 levels remained unchanged. β-actin served as a loading control. Quantitative analysis of these blots is presented in Fig. S1c (**c**) Representative immunofluorescence images (left) and quantification (right) of LC3B puncta in N2a cells under the indicated conditions. UA-36 treatment markedly increased the number of LC3B-positive puncta per cell, indicating enhanced autophagosome formation. Scale bar = 15 μm (*n* = 30 cells/group from three independent experiments) (**d**) Experimental timeline for autophagic flux assessment using chloroquine (CQ). Following FXN knockdown and UA-36 treatment, cells were co-treated with CQ (50 μM) for the final 4 h to block lysosomal degradation (**e**) Representative immunoblots (left) and quantification (right) of LC3B-II levels in cells treated as shown in (**d**). The further accumulation of LC3B-II upon CQ co-treatment in UA-36-treated cells confirms that UA-36 enhances autophagic flux rather than impairing lysosomal degradation (*n* = 5 independent experiments) (**f**) Experimental timeline for autophagy inhibition studies using CQ and 3-methyladenine (3-MA). Following FXN knockdown, cells were treated with UA-36 in the presence or absence of CQ (50 μM, last 4 h) or 3-MA (5 mM, autophagy initiation inhibitor) (**g**) Representative immunoblots showing FXN and autophagy markers under autophagy inhibition conditions. Pharmacological inhibition of autophagy with either CQ or 3-MA abolished UA-36-mediated restoration of FXN protein levels, demonstrating that UA-36 effects are autophagy-dependent. Quantitative analysis of these blots is presented in Fig. S1d (*n* = 3–6 independent experiments). Quantitative data in (**c**) and (**e**) are presented as mean ± SEM for *n* ≥ 4, or mean ± SD for *n* < 4. Statistical significance was determined using one-way ANOVA with Tukey’s post hoc test for multiple comparisons. *p < 0.05, **p < 0.01, ***p < 0.001, ****p < 0.0001; ns, not significant. CQ, chloroquine; 3-MA, 3-methyladenine; FXN, frataxin; KD, knockdown; h, hours; μM, micromolar; mM, millimolar

We next investigated whether UA-36 could rescue these deficits. Treatment of *Fxn*-KD cells with UA-36 (10 µM) for 24 h (Fig. 2a) significantly restored FXN protein content (Fig. 2b; Fig. S1c). Concurrently, UA-36 upregulated key autophagy proteins, including phosphorylated Unc-51-like autophagy activating kinase 1 (p-ULK1), Beclin-1, autophagy-related 5 (ATG5), and LC3B-II (Fig. 2b; Fig. S1c), and enhanced autophagosome formation, as visualized by increased LC3 puncta (Fig. 2c). However, the autophagy substrate sequestosome 1 (p62) remained unchanged (Fig. 2b; Fig. S1c). Moreover, UA-36 treatment did not affect mechanistic target of rapamycin (mTOR) phosphorylation (Fig. 2b; Fig. S1c), indicating its action is mTOR-independent. To distinguish between autophagic induction and flux, we employed chloroquine (CQ), which blocks lysosomal degradation [16]. Intriguingly, co-treatment with UA-36 and CQ led to a further accumulation of LC3B-II compared to CQ alone (Fig. 2d, e), demonstrating that UA-36 stimulates autophagic activity at a step prior to lysosomal fusion. To further delineate the underlying mechanism, we treated FXN-deficient N2a cells with autophagy inhibitors, CQ or 3-methyladenine (3-MA) [17]. Crucially, pharmacological inhibition of autophagy with CQ or 3-MA abolished UA-36-mediated rescue of FXN and autophagy markers, reducing FXN, p-ULK1, Beclin-1, and ATG5 levels while increasing p62 and LC3B-II accumulation consistent with autophagic flux blockade (Fig. 2f, g; Fig. S1d), confirming that UA-36’s effects require a functional autophagic pathway. These in vitro results suggest that UA-36 may exert a neuroprotective effect against cellular pathologies induced by frataxin deficiency.

### UA-36 rescues motor coordination and gait deficits in the YG8R mouse model of FA

**Fig. 3:**
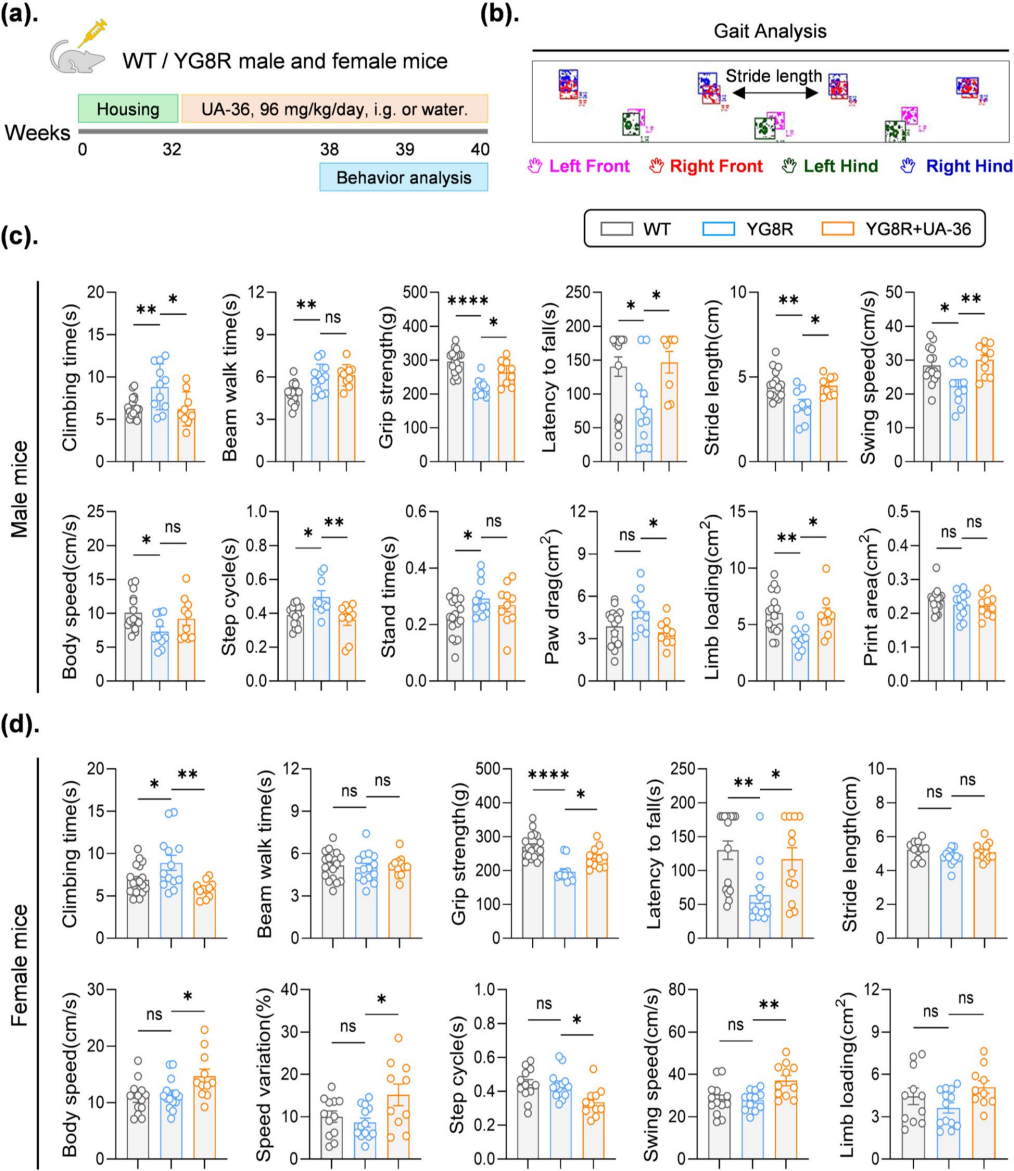
UA-36 improves motor coordination and gait performance in YG8R mice (**a**) Schematic diagram illustrating the experimental timeline for in vivo UA-36 treatment and behavioral assessments. WT and YG8R mice (male and female, 32 weeks old) were administered UA-36 (96 mg/kg/day, i.g.) or vehicle (water) for 8 consecutive weeks. Behavioral tests were performed at 39–40 weeks of age, followed by tissue collection for biochemical and histological analyses (**b**) Representative footprint patterns from gait analysis showing stride length and coordination in WT, untreated YG8R, and UA-36-treated YG8R mice. Colored paw prints indicate left front (blue), right front (red), left hind (green), and right hind (purple) limbs (**c**) Quantitative behavioral analyses in male mice (*n* = 9–19 per group) showing climbing time, grip strength, latency to fall (rotarod), beam walk time, and comprehensive gait parameters including stride length, swing speed, body speed, step cycle, limb loading, and paw drag area. UA-36 treatment significantly improved motor function across multiple parameters in male YG8R mice (**d**) Quantitative behavioral analyses in female mice (*n* = 9–19 per group) demonstrating UA-36-mediated improvements in climbing time, grip strength, rotarod performance, and selected gait parameters. Sex-dependent differences in treatment response were observed, with males showing greater gait normalization. All data are presented as mean ± SEM (*n* = 9–19 per group). Statistical significance was determined using one-way ANOVA with Tukey’s post hoc test for multiple comparisons. *p < 0.05, **p < 0.01, ***p < 0.001, ****p < 0.0001; ns, not significant. UA-36 treatment groups are color-coded as indicated in the schematic. i.g., intragastric administration; WT, wild-type; YG8R, Friedreich ataxia mouse model

To evaluate the therapeutic potential of UA-36 in vivo, we orally administered UA-36 (96 mg/kg, once daily) to 8-month-old YG8R mice for eight weeks, with behavioral assessments performed during the final two weeks of treatment (Fig. 3a). Consistent with established FA phenotypes, untreated YG8R mice exhibited significant motor deficits compared to wild-type (WT) controls, characterized by reduced grip strength, prolonged descent time in the pole test, and shorter latency to fall in the wire hanging test. Strikingly, UA-36 treatment significantly improved these parameters, effectively restoring muscle function and coordination toward wild-type levels, suggesting that UA-36 primarily enhances motor endurance and strength of mice (Fig. 3c).

Detailed gait analysis revealed that YG8R mice (male) display fundamental locomotor deficits characteristic of ataxia, including reduced body speed, decreased swing speed, shorter stride length, and abnormal paw loading. UA-36 treatment partially alleviated these gait abnormalities, leading to improved gait characteristics, including significantly increased swing speed and stride length. Additionally, the mice treated showed improved limb loading and reduced step cycle duration (Fig. 3b, c). These findings suggest that UA-36 not only enhances performance in structured motor tasks but also promotes better dynamic coordination during voluntary walking.

To explore the therapeutic potential of UA-36 across genders, we conducted an identical treatment and assessment paradigm in female YG8R mice. In the female cohort, UA-36 treatment was associated with improvements in grip strength, pole test performance, and wire hang latency. However, consistent with the results in males, the beam walk test did not show a significant change. Additionally, while gait analysis confirmed benefits in body speed and step cycle, no significant change in stride length was observed in the female groups (Fig. 3d).

Together, these results suggest that chronic oral administration of UA-36 improves motor coordination and gait function in both male and female YG8R mice. It should be noted that female mice exhibited less pronounced disease symptoms at this time point than males, which may limit the magnitude of the drug’s effect in this group. This disparity in symptom severity could potentially be attributed to the neuroprotective effects of estrogen, a factor that warrants further investigation. Therefore, male mouse samples were used for all subsequent mechanistic studies.

### UA-36 ameliorates systemic metabolic dysfunction and exhibits favorable pharmacokinetics

After validating that UA-36 rescues motor function, we next sought to determine whether its therapeutic impact extends to metabolic dysfunction characteristic of FA. Serum biochemical profiling revealed that YG8R mice exhibited a multifaceted systemic disturbance compared to wild-type controls, as evidenced by increased oxidative stress (elevated malondialdehyde, MDA) and altered markers of kidney function, hepatic function, and protein metabolism (urea, globulin, and albumin/globulin ratio). Interestingly, treatment with UA-36 robustly ameliorated this systemic dysregulation by significantly reducing triglycerides (TG) and MDA levels and improving key hepatic and protein balance markers (aspartate aminotransferase-to-alanine aminotransferase ratio, AST/ALT; and albumin/globulin ratio) in YG8R-mice (Fig. S2a). These results suggest that UA-36 exerts a broad therapeutic effect on the metabolic pathophysiology associated with FA. Critically, pharmacokinetic profiling revealed that oral administration of UA-36 achieved significantly higher plasma concentrations and superior bioavailability compared to UA (Tables S1-S4). This improved systemic exposure provides a robust pharmacokinetic foundation for the enhanced multi-system efficacy observed with UA-36 treatment.

### UA-36 modulates tissue-specific redox and inflammatory markers in the nervous system

We next performed a detailed biochemical analysis to determine whether UA-36 could ameliorate tissue-specific redox and inflammatory imbalances within the central nervous system. In the cerebellum, YG8R mice exhibited significant oxidative stress and mitochondrial depletion, characterized by elevated reactive oxygen species (ROS) and a marked reduction in mitochondrial DNA (mtDNA) copy number. Concurrently, a distinct inflammatory profile was evident, with increased levels of tumor necrosis factor-alpha (TNF-α) and interleukin-10 (IL-10). Treatment with UA-36 effectively restored cerebellar homeostasis, significantly reducing ROS, increasing mtDNA copy number, and elevating nitric oxide (NO) and H₂O₂ levels. Furthermore, UA-36 normalized the aberrant cytokine profile, reducing TNF-α and IL-10 (Fig. S2b). UA-36 treatment uniquely and selectively restored the concentrations of these cytokines and mitochondrial-related markers (Fig. S2b), indicating a targeted, compartment-specific immunomodulatory action. Together, these multi-tissue analyses demonstrate that UA-36 corrects redox and inflammatory imbalance in the cerebellum.

### UA-36 prevents neurodegeneration and peripheral tissue damage in YG8R mice

**Fig. 4:**
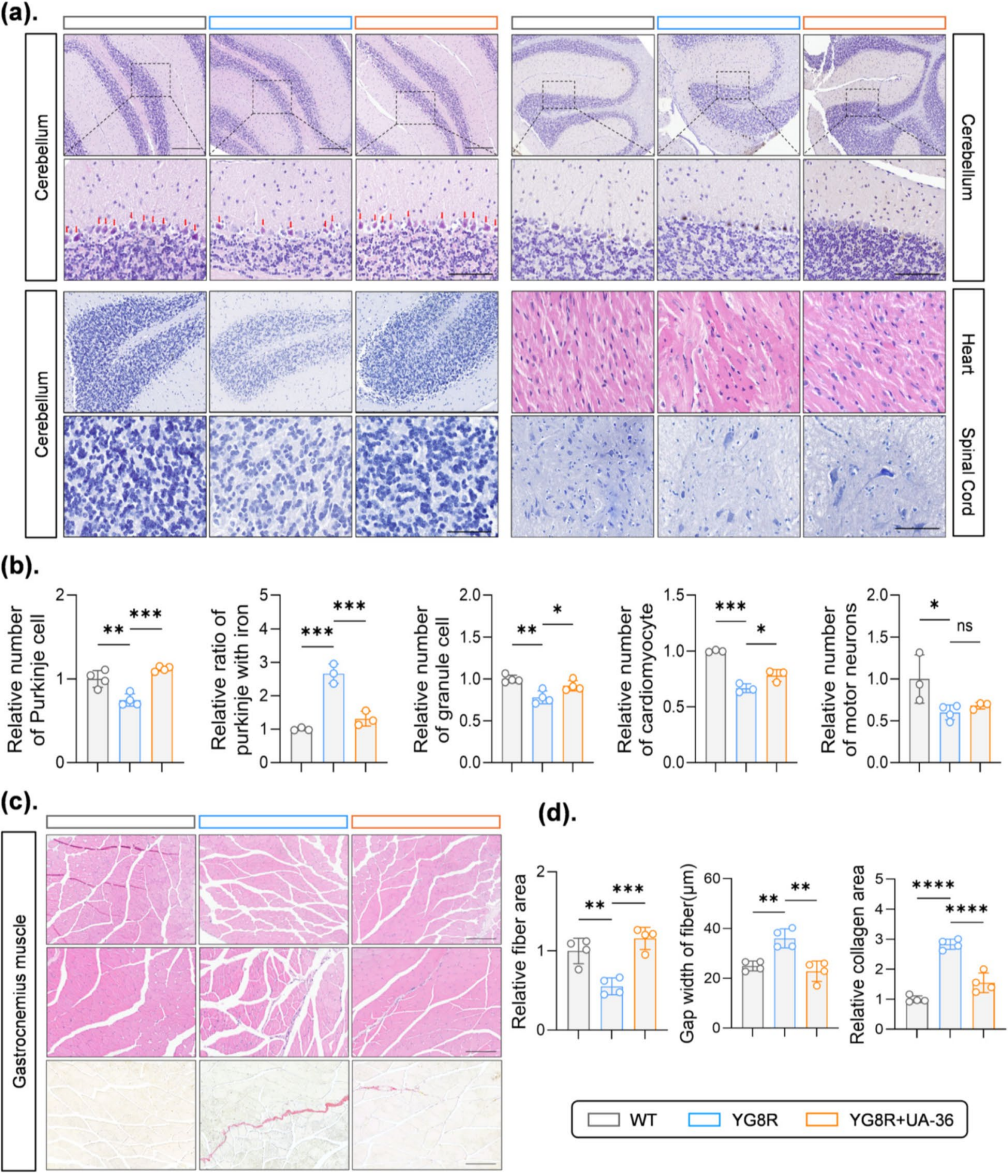
UA-36 alleviates histopathological abnormalities across multiple tissues in YG8R mice (**a**) Representative histological images (left) and quantitative analysis (right) of cerebellar pathology in WT, untreated YG8R, and UA-36-treated YG8R mice. Hematoxylin and eosin (HE) staining shows Purkinje cell morphology and density (zoom-in panels highlight individual Purkinje cells). Prussian blue staining reveals iron accumulation (blue deposits). Nissl staining displays granule cell layer density. UA-36 treatment significantly preserved Purkinje cell numbers, reduced pathological iron accumulation, and maintained granule cell density. Scale bars: 60 μm for Purkinje cell-HE (overview); 30 μm for Purkinje cell-HE (zoom-in), Prussian blue, and Nissl staining (zoom-in) (**b**) Quantification of cerebellar histopathology: Purkinje cell count per field, iron deposit area fraction, and granule cell density (*n* ≥ 4 mice per group) (**c**) Representative histological images of peripheral tissues. HE-staining of heart tissue shows cardiomyocyte morphology and organization. Nissl staining of the spinal cord anterior horn displays motor neuron integrity. Muscle tissue sections show inter-fiber gap area (indicating atrophy) and collagen deposition (Sirius red staining) with corresponding fiber area quantification. UA-36 treatment attenuated cardiac pathology, preserved spinal cord motor neurons, and reduced muscle atrophy and fibrosis. Scale bars: 60 μm for Heart-HE; 50 μm for Spinal cord-Nissl; 180 μm for inter-fiber gaps and collagen deposition; 360 μm for muscle fiber area overview (**d**) Quantification of peripheral tissue pathology: cardiac fibrosis area, spinal cord motor neuron count, muscle inter-fiber gap area, collagen deposition area, and mean muscle fiber cross-sectional area (*n* ≥ 4 mice per group). All quantitative data are presented as mean ± SEM (*n* ≥ 4 mice per group). Statistical significance was determined by one-way ANOVA with Tukey’s post-hoc test. *p < 0.05, **p < 0.01, ***p < 0.001, ****p < 0.0001; ns, not significant. Scale bars are indicated for each staining type. HE, hematoxylin and eosin; PB, Prussian blue; SR, Sirius red

Following the demonstration of functional and biochemical improvements, we next examined whether UA-36 could prevent systemic tissue damage characteristic of FA. Histopathological analysis of YG8R mice revealed pronounced cerebellar neurodegeneration, evidenced by increased iron-laden Purkinje cells and neuronal loss (Fig. 4a, b). UA-36 treatment substantially reduced cerebellar iron deposition and restored the density of both Purkinje and granule cell layers. In the heart, UA-36 significantly alleviated cardiomyocyte hypertrophy. In the spinal cord, however, UA-36 treatment did not increase motor neuron counts substantially compared to untreated YG8R mice (Fig. 4a, b), suggesting that its primary protective effects may be more pronounced in other tissue compartments. Concurrently, histological analysis of skeletal muscle (gastrocnemius) demonstrated that UA-36 treatment improved fiber atrophy and reduced pathological inter-fiber spacing (Fig. 4c, d). Notably, Sirius Red staining revealed that UA-36 significantly attenuated interstitial fibrosis, as evidenced by a marked reduction in collagen deposition compared to untreated YG8R mice. These findings indicate that UA-36 confers multi-tissue protection, effectively targeting both central neurological degeneration and the systemic fibrotic and musculoskeletal manifestations of FA.

**Fig. 5:**
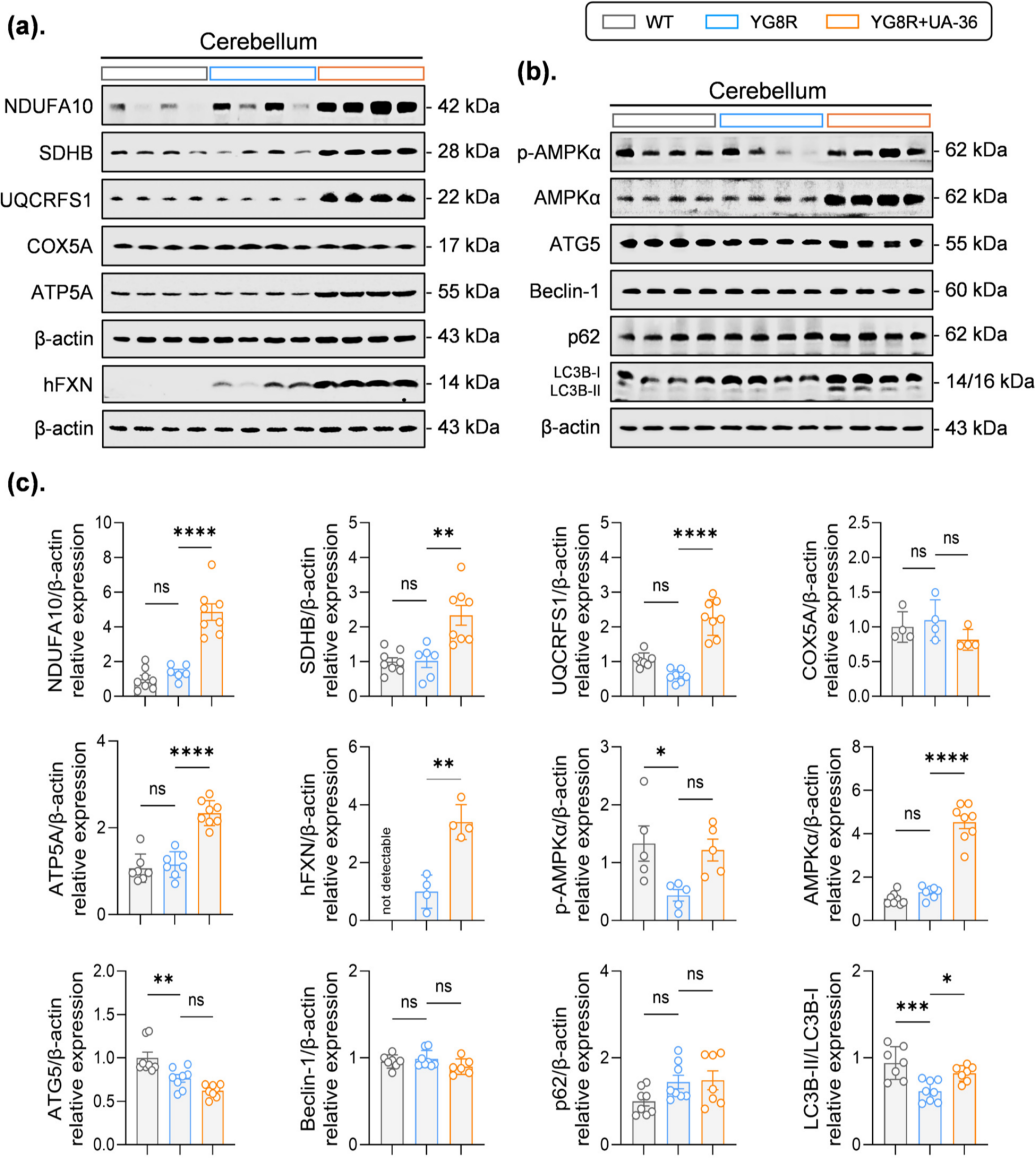
UA-36 restores dysregulated mitochondrial and autophagic signaling pathways in the cerebellum of YG8R mice (**a**) Representative immunoblots of cerebellar tissue lysates from WT, untreated YG8R, and UA-36-treated YG8R mice. Protein levels were assessed for human frataxin (hFXN), and mitochondrial electron transport chain subunits (NDUFA10, SDHB, UQCRFS1, ATP5A, COX5A). β-actin served as a loading control (**b**) Representative immunoblots of energy-sensing kinases (p-AMPKα, total AMPKα), and autophagy-related markers (LC3B-I/II, ATG5, Beclin-1, p62). β-actin served as a loading control (**c**) Densitometric quantification of protein expression levels normalized to β-actin (*n* = 4–8 mice per group). UA-36 treatment significantly increased hFXN levels in YG8R mice and restored multiple electron transport chain subunits (NDUFA10, SDHB, UQCRFS1, ATP5A) toward WT levels, while COX5A remained unchanged. Energy-sensing signaling was enhanced, as evidenced by increased p-AMPKα/AMPKα ratio. Autophagic flux was increased, as indicated by a significant increase in the LC3B-II/LC3B-I ratio, whereas ATG5, Beclin-1, and p62 levels showed no significant differences compared with untreated YG8R mice. All quantitative data are presented as mean ± SEM for *n* ≥ 4, or mean ± SD for *n* < 4. Statistical significance was determined by one-way ANOVA with Tukey’s post-hoc test. *p < 0.05, **p < 0.01, ***p < 0.001, ****p < 0.0001; ns, not significant. Exact p-values and n numbers are provided in the Source Data. hFXN, human frataxin; ETC, electron transport chain; AMPK, AMP-activated protein kinase

We next investigated the molecular mechanisms underlying UA-36’s neuroprotective effects, focusing on the cerebellum, a primary site of FA pathology. Molecular profiling of YG8R mice revealed extensive deficits in redox balance, mitochondrial integrity, and autophagic flux (Fig. 5a, b, and c; Fig. S3c). Specifically, YG8R mice exhibited reduced antioxidant defenses (SOD2, GPX4, NFS1), impaired mitochondrial biogenesis (PGC-1α, Tfam), and compromised electron transport chain function (UQCRFS1). These deficits were accompanied by suppressed energy-sensing (p-AMPKα) and autophagy markers (ATG5 and LC3B-II). In contrast, SIRT1 protein levels were elevated, likely reflecting a compensatory response to stress.

Notably, UA-36 treatment significantly enhanced the level of human frataxin (hFXN) and comprehensively mitigated these downstream pathological changes (Fig. 5a, b, and c). Western blot analysis demonstrated that UA-36 administration significantly restored redox homeostasis by upregulating key antioxidants (HO-1, GPX4, NFS1) and rescued mitochondrial quality control by enhancing mitophagy (PINK1, PARKIN) and biogenesis (PGC-1α, OPA1) markers (Fig. S3c). Furthermore, UA-36 reactivated autophagic flux, as evidenced by increased levels of ATG5, p-AMPKα, and LC3B-II (Fig. 5a, b, and c), while normalizing elevated SIRT1 levels (Fig. S3c). These findings demonstrate that UA-36 concurrently mitigates the core pathologies of FA by integrating the rescue of redox, mitochondrial, and proteostatic pathways.

### Proteomic profiling identifies autophagy restoration as a key mechanism of UA-36 action

We next performed TMT-based quantitative proteomics on cerebellar tissue to define the global proteomic signature of FA pathology and the systemic impact of UA-36 treatment (Fig. S4a). Comparative analysis revealed profound proteome dysregulation in YG8R mice, with 1,895 proteins significantly altered compared to wild-type (WT) controls (847 upregulated, 1,048 downregulated (Fig. S4b). Kyoto Encyclopedia of Genes and Genomes (KEGG) pathway analysis of these alterations defined the pathological signature: upregulated proteins were enriched in translation, glycolysis, and cell division, whereas downregulated proteins were strikingly depleted in autophagy, mitochondrial organization, ubiquitin-mediated proteolysis, and synaptic function, establishing failure in mitochondrial homeostasis imbalance, abnormal autophagy, and metabolic abnormalities as a central hallmark of FXN-deficinet pathology (Fig. S4c, d).

**Fig. 6:**
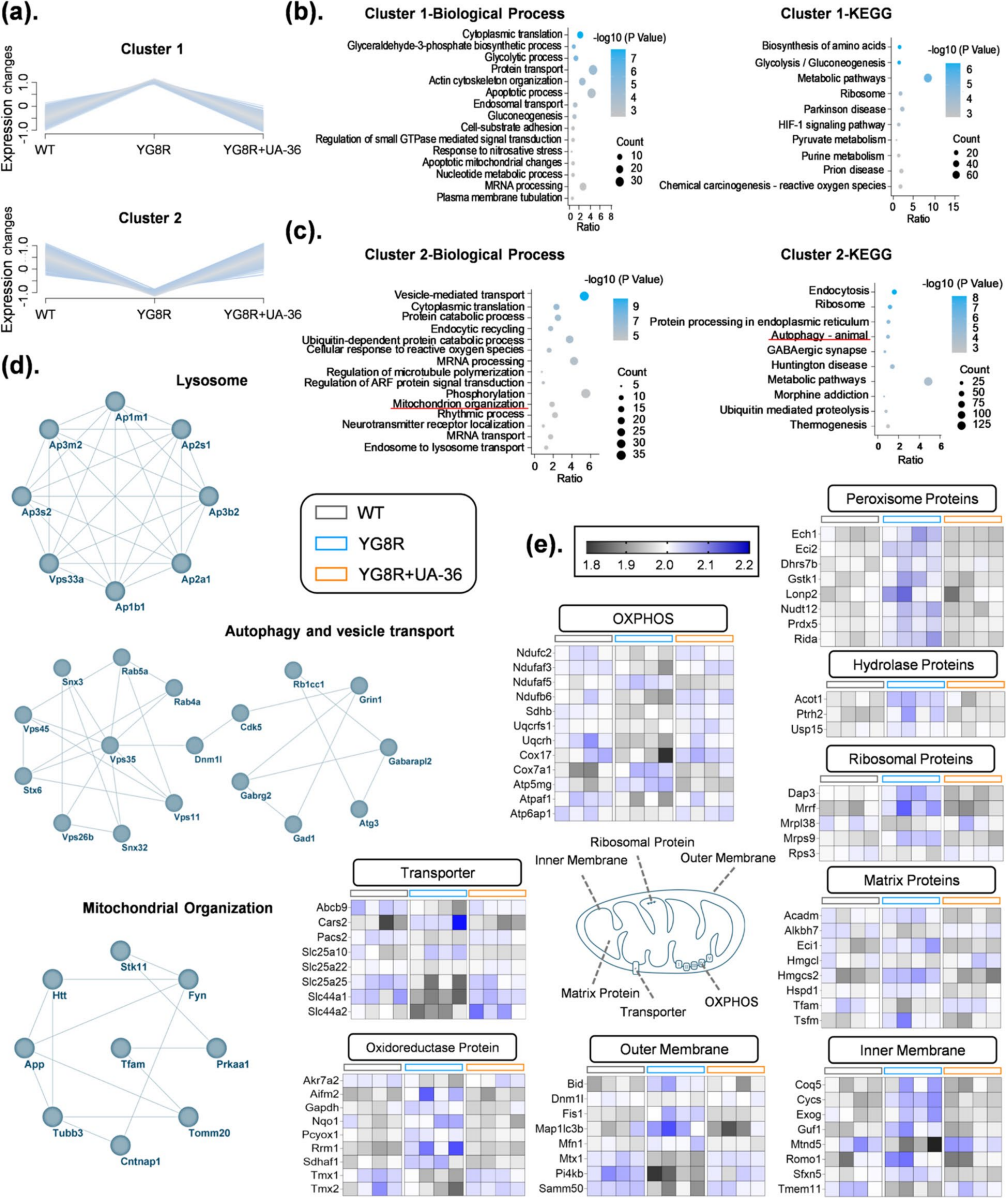
Proteomic analysis identifies suppression of the autophagy pathway in FXN-deficient cerebellum and its restoration by UA-36 (**a**) Hierarchical clustering heatmap of quantitative proteomic profiling performed on cerebellar tissue from WT, YG8R, and YG8R + UA-36 mice (*n* = 4 mice per group). Protein expression levels are color-coded from low (blue) to high (red). Two major clusters were identified: Cluster 1 (proteins downregulated by UA-36 treatment) and Cluster 2 (proteins upregulated by UA-36 treatment), revealing distinct expression patterns across experimental groups (**b**) Gene Ontology (GO) and KEGG pathway enrichment analysis of Cluster 1 proteins. This cluster showed significant enrichment for pathways associated with apoptosis, oxidative stress, and nitrosative stress, consistent with FX N-deficiency-driven pathology attenuated by UA-36 treatment (**c**) GO and KEGG enrichment analysis of Cluster 2 proteins. This cluster demonstrated robust enrichment of proteins involved in autophagy, vesicular trafficking, lysosomal function, and mitochondrial organization, indicating pathway-level rescue by UA-36 treatment in YG8R mice (**d**) Protein–protein interaction (PPI) network analysis of key proteins identified in the corresponding biological processes. Network nodes represent proteins, and edges represent functional or physical interactions. The analysis reveals coordinated interaction networks among autophagy-related proteins (autophagy and vesicle transport cluster) and mitochondrial organization proteins (mitochondrial organization cluster), both of which are modulated by UA-36 (**e**) Heatmap analysis of mitochondrial protein expression profiles across experimental groups. Expression patterns of individual mitochondrial proteins are shown, demonstrating the influence of UA-36 treatment on mitochondrial protein abundance in the FXN-deficient cerebellum. Proteomic analysis was performed on cerebellar tissue from *n* = 4 mice per group. Protein identification and quantification were performed using LC–MS/MS. GO and KEGG enrichment analyses were conducted using DAVID Bioinformatics Resources. PPI networks were constructed using the STRING database

Remarkably, treatment with UA-36 induced a substantial correction of this dysregulated proteome. Direct comparison showed that UA-36 normalized the expression of 1,153 proteins altered in YG8R mice. Unsupervised hierarchical clustering of these differentially expressed proteins clearly segregated the experimental groups into two primary clusters based on their expression trends, visually demonstrating that UA-36 treatment shifts the YG8R proteomic profile toward a WT-like state (Fig. 6a, Fig. S3a, b).

Cluster 1 comprised proteins that were upregulated in YG8R mice and subsequently decreased by UA-36 treatment; functional enrichment of this cluster revealed the downregulation of pathways associated with cellular stress and disorganization, specifically apoptotic processes and responses to oxidative and nitrosative stress (Fig. 6b). Conversely, Cluster 2 consisted of proteins downregulated in the disease model that were significantly increased by UA-36 (Fig. 6c). These proteins were primarily involved in essential homeostatic pathways, including mitochondrion organization, autophagy, vesicle transport, and lysosomal function (Fig. S3d). Together, these data indicate that UA-36 exerts a dual-action therapeutic effect by suppressing proteotoxic stress while restoring vital organelle and transport functions.

To delineate the core mechanistic axis, we performed focused analysis on rescued pathways. It confirmed a concerted upregulation of the entire autophagic-lysosomal system, including key components for autophagosome formation (Gabarap12), vesicle tethering (Vps11, Vps45), endosomal sorting (Vps26b), and lysosomal function (Fig. 6d). Furthermore, UA-36 restored a network of proteins governing mitochondrial organization, dynamics (DNM1, OPA1), and transport (Fig. 6d and e), effectively re-establishing overall mitochondrial protein homeostasis.

Collectively, our proteomic profiling provides systems-level validation that UA-36 alleviates FA pathology by counteracting the pathological upregulation of anabolic processes, reactivating stalled autophagic-lysosomal flux, and restoring mitochondrial proteostasis, thereby mitigating the accumulated oxidative and proteotoxic stress that characterizes FA.

## Discussion

The progressive neurodegeneration of FA presents a critical therapeutic challenge, with its pathogenesis rooted in the triad of mitochondrial failure, oxidative stress, and impaired cellular clearance [18]. While the role of autophagy in this cascade is increasingly recognized [19], its therapeutic exploitation has remained limited. Our study identifies a precise blockade of autophagic flux, specifically a failure to complete autophagy despite enhanced initiation as a central, actionable node in frataxin (FXN)-deficient pathology. We introduce UA-36, a novel Urolithin A derivative, as a potent therapeutic agent that restores this flux. By demonstrating that UA-36 rescues mitochondrial integrity, reduces ferroptotic damage, and improves motor function, our findings establish targeted autophagy restoration as a viable disease-modifying strategy for FA.

A key advancement of our work is the rational design of UA-36 to overcome the pharmacokinetic limitations of its parent compound, Urolithin A (UA) [15, 20]. While UA is a known mitophagy inducer, its therapeutic potential is constrained by suboptimal bioavailability [13, 21]. UA-36 was engineered to bridge this gap. Our data reveal that UA-36 not only retains the beneficial properties of UA but also exhibits superior efficacy in more robustly restoring FXN protein levels, normalizing mitochondrial networks, and rescuing behavioral deficits in the YG8R mouse model. Proteomic analysis confirms this systems-level impact, showing that UA-36 orchestrates comprehensive cellular reprogramming by downregulating apoptotic pathways while upregulating networks essential for vesicle transport, lysosomal function, and mitochondrial organization.

Our findings suggest that mitochondrial dysfunction and oxidative stress in FA are fundamentally linked to impaired autophagic flux [22]. UA-36 is a promising lead compound that facilitates the clearance of dysfunctional organelles and metabolic debris. Notably, this effect is independent of mTOR signaling, as evidenced by stable mTOR phosphorylation levels. Instead, UA-36 acts by restoring AMPK activity, a master metabolic sensor [23] reported to be dysregulated in FXN-deficient states [24, 25]. This mTOR-independent mechanism is of significant clinical interest; targeting upstream metabolic sensors may offer a more specific and tolerable therapeutic approach than broad mTOR inhibition, which is frequently associated with toxic side effects in neurodegenerative contexts [26].

In parallel, the observation that UA-36 significantly upregulates FXN mRNA indicates that the compound enhances frataxin through both transcriptional and post-translational modulation. We propose that the restoration of autophagic flux serves as the primary initiating event; by clearing damaged mitochondria and alleviating chronic stress, UA-36 creates a permissive environment for nuclear-encoded gene expression. This reactivation is followed by the stabilization of PGC-1α, a master regulator of mitochondrial biogenesis [27]. We posit that this recovery triggers a retrograde signaling pathway, a form of nuclear-mitochondrial crosstalk that positively regulates the transcription of nuclear genes, including the hFXN transgene.

Notably, several mitochondrial, antioxidant, and quality-control proteins increased beyond WT levels following UA-36 treatment. We interpret this coordinated upregulation not as an off-target effect, but as a therapeutically beneficial potentiation of endogenous stress-response pathways. In FA, baseline levels of these proteins are insufficient to counteract chronic mitochondrial dysfunction. UA-36 likely elevates them above the pathological threshold required to restore homeostasis, a principle well established in neurodegenerative therapeutics [28]. Prominently, this potentiation occurred alongside clear improvements in molecular, histological, and behavioral outcomes without toxicity, supporting its role as an on-pathway, disease-modifying mechanism.

The therapeutic efficacy of UA-36 stems from its comprehensive impact on both central and peripheral pathologies. A primary driver of this protection is the regulation of iron homeostasis and the mitigation of oxidative stress, both of which are recognized hallmarks of FA pathology [22]. By reducing pathological iron accumulation and restoring redox balance, UA-36 preserves cerebellar Purkinje cell integrity and ameliorates degenerative changes in cardiac and skeletal muscle. This integrated multi-tissue rescue is functionally validated by the normalization of gait parameters, specifically increased swing speed and stride length [10, 29]. Furthermore, the restoration of mitochondrial function and energy-sensing pathways within related motor circuits likely underlies the enhanced grip strength and coordination observed in our behavioral assessments [30], collectively demonstrating the systemic disease-modifying potential of UA-36.

From a clinical translation perspective, UA-36 represents a highly promising lead compound due to its favorable pharmacokinetic profile and oral bioavailability. Crucially, our work shifts the therapeutic paradigm from general mitochondrial support toward the precision correction of autophagic flux. Despite these findings, several limitations remain. The precise primary molecular target of UA-36 and the exact role of energy-sensing pathways remain to be elucidated. Furthermore, while the YG8R model recapitulates core FA features, the long-term efficacy and the lack of significant improvement in certain parameters among female cohorts, potentially due to the protective influence of estrogen or a milder baseline phenotype, warrant further longitudinal and sex-specific investigation.

In summary, our study identifies impaired autophagic flux as a core pathogenic mechanism in FA and demonstrates that UA-36 effectively reverses this defect. By rescuing mitochondrial and motor function in vivo through a mechanism distinct from traditional support strategies, these findings validate autophagy restoration as a viable therapeutic strategy. This positions UA-36 as a promising lead candidate for the treatment of FA and related neurodegenerative disorders.

## Material and methods

### Animals

The experimental model used was the YG8R transgenic mouse line (B6.Cg-*Fxn* tm1Mkn Tg(FXN)YG8Pook/J, Stock No: 012253; The Jackson Laboratory, Bar Harbor, ME), a homozygous *Fxn* knockout with a human *FXN* transgene containing 90–190 GAA repeats on a C57BL/6J background. These mice exhibit a progressive Friedreich’s ataxia phenotype, with onset between 8 and 10 months. For this study, 32-weeks-old male and female mice were used. Wild-type C57BL/6J mice (Stock No: 000664; Guangdong Medical Laboratory Animal Center, Foshan, China), age- and sex-matched littermates, verified by PCR genotyping, served as controls, as suggested by Jackson Laboratory (https://www.jax.org/strain/012253).

Mice were housed in a specific pathogen-free (SPF) environment maintained at 21 ± 1 °C, 50 ± 10% humidity, and a 12:12 light–dark cycle (lights on 07:00–19:00). Nesting material and PVC shelters were provided. Mice were given ad libitum access to standard chow (LabDiet 5053) and autoclaved tap water via sipper tubes.

All experimental protocols were approved by the Peking University Shenzhen Graduate School Institutional Animal Care and Use Committee (approval number: 11110). Animal care and use adhered to AAALAC International guidelines.

### Drug treatment and tissue collection

UA-36, a novel urolithin A derivative with improved water solubility and bioavailability, was synthesized and used in the experiment. The pharmacokinetic parameters in mice for UA-36 and the identification data are presented in the supplementary data. The dosage was converted from molar doses used in mouse experiments and dissolved in purified water [31].

Mice were randomly assigned to three groups: Wild-Type (WT), YG8R, and YG8R + UA-36. Animals in the YG8R + UA-36 group received UA-36 at a dose of 96 mg/kg by oral gavage once daily for 8 consecutive weeks, whereas the other two groups of mice received an equivalent volume of purified water. All treatments were administered at the same time each day to minimize circadian variability.

Behavioral assessments were conducted during the final two weeks of the treatment period. At the end of the experiment, mice were euthanized, and tissues, including the cerebellum, spinal cord, heart, and gastrocnemius muscle, were rapidly dissected. Blood samples were collected for serum preparation. All tissue and serum samples were immediately snap-frozen in liquid nitrogen and stored at −80 °C until further biochemical and molecular analyses. For all non-behavioral analyses, we restricted our investigations to the samples from male mice.

### Behavior analysis

The behaviors tests, including the Pole Test, Beam Walk Test, Grip Strength Test, Wire Hanging Test, and Gait Analysis, were performed as previously described [32] with modifications. Detailed methods are described in the supplementary data.

### Histological analysis

Histological analysis, including Hematoxylin and Eosin (H&E) [33, 34], Nissl, Prussian Blue Iron [35–37] and Sirius Red [38], was performed as previously described and according to the protocol developed in our lab [32, 39, 40]. Detailed methods are described in the supplementary data.

### Proteomics

Proteomic analysis was performed as previously described [40]. The experimental workflow, including the sample groups (WT, YG8R, and YG8R + UA-36; *n* = 4 per group) and the TMT labeling procedure, is summarized in **Fig. S**4a. Detailed methods are provided in the Supplementary Data.

### Bioinformatics analysis

Analyze the relative abundance of differentially expressed proteins within each group using heatmaps and utilize David’s bioinformatics resources (https://david.ncifcrf.gov/). Perform cluster analysis to demonstrate corresponding biological processes and KEGG enrichment pathways. Use Molecular Complex Detection (MCODE) to identify dense regions of protein–protein interactions (PPI) and then use Hiplot (https://hiplot-academic.com/) to visualize the results.

### Enzyme-Linked Immunosorbent Assay (ELISA)

ELISA was performed as described previously [41–43]. Detailed methods are described in the supplementary data.

### Quantitative real-time PCR

Total RNA was isolated from cerebellar tissue using TRIzol reagent (RC112, Vazyme, China). cDNA was then generated with the HiScript IV All-in-One Ultra RT SuperMix Kit (R433, Vazyme, China). qRT-PCR was carried out using SupRealQ Ultra Hunter SYBR qPCR Master Mix (Q713, Vazyme, China) in accordance with the manufacturer protocol, with each sample analyzed in at least duplicate. The relative transcript levels were normalized to β-actin expression. The primary information is as follows.

Primer sequences for p62.

(F) GGGAACACAGCAAGCTCATC

(R) TGTCAACCTCAATGCCTAGAG.

Primer sequences for β-actin.

(F) ACCAGAGGCATACAGGGACA

(R) CTAAGGCCAACCGTGAAAAG.

### Mitochondrial copy number detection

After extracting genomic DNA from cerebellar tissue and cell samples using the TIANamp Genomic DNA kit (DP304, TIANGEN, China), qPCR detection was performed using TransScript Tip Green qPCR SuperMix (AQ601, Transgen Biotech, China). The primary information is as follows.

Primer sequences for the mitochondrial segment:

(F) GCCAGCCTGACCCATAGCCATAAT

(R) GCCGGCTGCGTATTCTACGTTA.

Primer sequences for the single-copy nuclear control:

(F) TTGAGACTGTGATTGGCAATGCCT

(R) CCAGAAATGCTGGGCGCTCACT.

Relative abundance was normalized to the nuclear control.

### Serum analysis

MDA in serum was detected by Lipid Peroxidation MDA Assay Kit (S0131, Beyotime, China). According to the manufacturer instructions, the serum was diluted and reacted with the TBA detection solution, and absorbance was measured at 535nm. The remaining serum indicators were detected using a fully automated biochemical analyzer (Mindray, China).

### GSH/H_2_O_2_/NO measurement

These were analyzed using a commercially available kit (S0053/S0021M/S0038, Beyotime, China) according to the manufacturer’s testing protocol.

### Cell culture and treatment

Mouse neuroblastoma N2a cells (authenticated by short tandem repeat (STR) analysis and tested negative for mycoplasma contamination) were cultured in Dulbecco’s Modified Eagle Medium (DMEM) medium (C11995500BT, Gibco, USA) (Gibco, USA) containing 10% fetal bovine serum in a humidified incubator at 37 °C and 5% CO2. The UA-36 dosage was selected using the Cell Counting Kit-8 (HY-K0301, MedChemExpress, USA), and the treatment time was based on previous studies [44]. The processing conditions for 3-MA and CQ are also based on prior research [39, 44].

Two plasmids were used in the experiment: one for Frataxin knockdown (TG514670, Origene, USA) and the other for mCherry-GFP-LC3B labeling of intracellular LC3B. According to the manufacturer’s instructions, Lipofectamine 3000 (L3000015, Thermo, USA) was used for transfection, and cells were collected 72 h later.

The ROS probe (CA1420, Solarbio, China) used in the experiment was used according to the manufacturer’s instructions and imaged by laser confocal microscopy.

### Western blot

Western blots were performed as previously described [39, 45–47]. Detailed methods are described in the supplementary data.

### Statistical analysis

Data are presented as mean ± standard deviation (SD) or mean ± standard error of the mean (SEM), as indicated in the corresponding figure legends and determined by sample size (*n* ≤ 4: SD; *n* > 4: SEM). For all bar charts, individual data points are shown as hollow circles to display the distribution of biological replicates. Statistical analyses were performed using GraphPad Prism 8. The exact number of biological replicates (n), definition of n, and the statistical test applied for each experiment are specified in the individual figure legends.

Comparisons between two groups were performed using unpaired two-tailed Student’s t-tests. Comparisons among three or more groups were performed using one-way analysis of variance (ANOVA), followed by Tukey’s post hoc test for multiple comparisons. Where applicable, behavioral assessments and cell counting were conducted by investigators blinded to the experimental groups. A *P* value < 0.05 was considered statistically significant and is denoted as* P* < 0.05, *P < 0.01, **P < 0.001, ***P < 0.0001, ****P < 0.00001, ns, not significant.

## Supplementary Information

Supplementary Material 1.

Supplementary Material 2.

## Data Availability

All other data generated or analyzed during this study are included in this published article and its supplementary information files. The mass spectrometry proteomics data have been deposited in the ProteomeXchange Consortium via the iProX partner repository with the dataset identifier PXD075931. The data can be accessed at the following link: https://www.iprox.cn/page/SSV024.html;url=1774002420311IK52. Any additional data supporting the findings of this study are available from the corresponding author upon reasonable request.
