# Supplementary material for: Autophagy activation by urolithin-a derivative UA-36 mitigates Friedreich’s ataxia pathologies induced by frataxin deficiency
Source: Mol Biomed. 2026 Jun 4;7:82. doi: 10.1186/s43556-026-00457-w (PMC13237351; doi:10.1186/s43556-026-00457-w)
Supplement: Supplementary file 1 — Supplementary Material 1. [file 43556_2026_457_MOESM1_ESM.docx]

**Supplementary Data**

**Autophagy Activation by Urolithin-A Derivative UA-36 Mitigates Friedreich's Ataxia Pathologies Induced by Frataxin Deficiency**

**Authors:** Qichao Gong¹,², Tiansu Liu²,³, Xiao Han²,⁴*, Ruiming Zhang⁵, Xinlei Liu⁵, Bocheng Xiong², Tahir Ali¹, Jianxiang Huang⁷, Yongmei Xie⁵#, Shupeng Li¹,⁶#, Xifei Yang²#

**Affiliations:**

1. State Key Laboratory of Chemical Oncogenomics, School of Chemical Biology and Biotechnology, Peking University Shenzhen Graduate School, Shenzhen, 518055, China.
2. Shenzhen Key Laboratory of Modern Toxicology, Shenzhen Medical Key Discipline of Health Toxicology (2020-2024), Shenzhen Center for Disease Control and Prevention, Shenzhen, 518055, China.
3. School of Public Health, Key Laboratory of Environmental Pollution Monitoring and Disease Control, Ministry of Education, Guizhou Medical University, Guiyang 561113, China.
4. Department of Toxicology, School of Public Health, Shanxi Medical University, Taiyuan 030001, Shanxi, China.
5. State Key Laboratory of Biotherapy and Cancer Center, West China Hospital, Sichuan University, and Collaborative Innovation Center of Biotherapy, Chengdu, 610041, PR China.
6. Functional Microbiology Research and Development Center, Research Institute of Tsinghua University in Shenzhen, Shenzhen, Guangdong 518055, China.
7. Institute for Future Human Habitats, Tsinghua University Shenzhen International Graduate School, Shenzhen, Guangdong Province, China.

**Corresponding authors:** Yongmei Xie (xieym@scu.edu.cn), Shupeng Li (lisp@pku.edu.cn), Xifei Yang (xifeiyang@gmail.com)

*These authors contributed equally to this work.


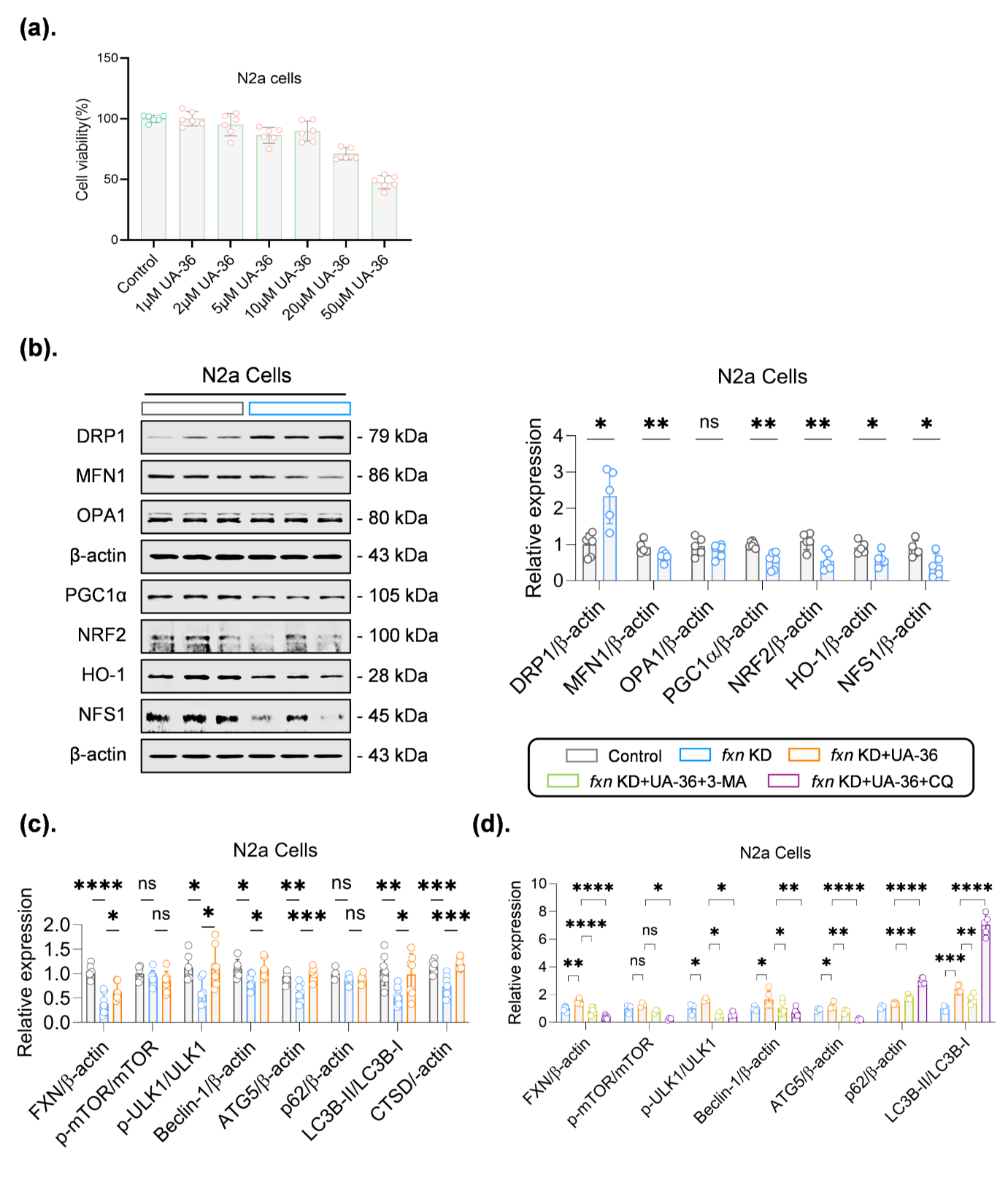


**Fig. S1** FXN deficiency induces mitochondrial dysfunction and autophagy impairment in N2a cells (a) Dose-response curve of N2a cells treated with varying concentrations of UA-36 (1–50 µM) for 24 h, measured by CCK-8 assay (b) Representative immunoblots (left) and corresponding densitometric quantification (right) revealing that FXN knockdown significantly alters mitochondrial dynamics, as evidenced by dysregulation of DRP1 and MFN1, and induces oxidative stress-related responses (NRF2, HO-1, NFS1) and PGC-1α expression. β-actin served as a loading control (n = 4–6 independent experiments) (c) Densitometric quantification of immunoblots from main Fig. 2b showing autophagy signaling pathways in FXN-deficient cells, characterized by altered ULK1 phosphorylation and aberrant expression of core autophagy and lysosomal markers (Beclin-1, ATG5, p62, LC3B-II, and CTSD), while mTOR phosphorylation remained unchanged. β-actin served as a loading control (n = 4–6 independent experiments) (d) Densitometric quantification of immunoblots from main Fig. 2g, demonstrating marked disruption of autophagy signaling in FXN-deficient cells (n = 4–6 independent experiments). All quantitative data in (b-d) are presented as mean ± SEM for n ≥ 4, or mean ± SD for n < 4. Statistical significance was determined by unpaired Student's t-test for (b) and one-way ANOVA with Tukey's post-hoc test for (c-d). *p < 0.05, **p < 0.01, ***p < 0.001, ****p < 0.0001; ns, not significant. DRP1, dynamin-related protein 1; MFN1, mitofusin 1; NRF2, nuclear factor erythroid 2-related factor 2; HO-1, heme oxygenase-1; NFS1, cysteine desulfurase; PGC-1α, peroxisome proliferator-activated receptor gamma coactivator 1-alpha; ULK1, Unc-51-like kinase 1; ATG5, autophagy-related protein 5; LC3B, microtubule-associated protein 1 light chain 3 beta; CTSD, cathepsin D; mTOR, mammalian target of rapamycin.


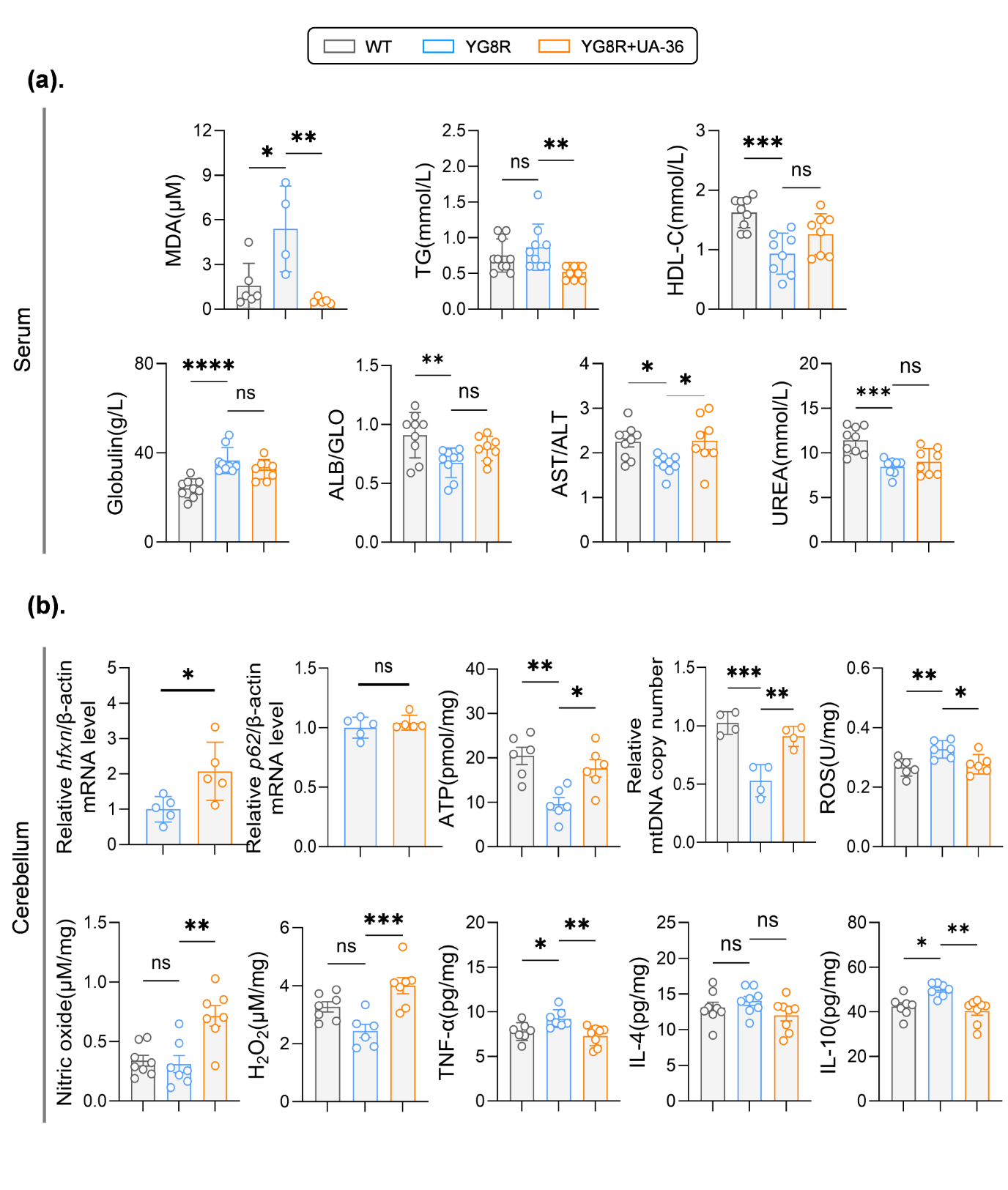
**Fig. S2** UA-36 improves mitochondrial function, redox homeostasis, and systemic biochemical parameters in YG8R mice (a) Serum biochemical analyses from WT, untreated YG8R, and UA-36-treated YG8R mice revealing that UA-36 treatment partly normalized metabolic and tissue injury markers, including malondialdehyde (MDA), triglycerides (TG), high-density lipoprotein cholesterol (HDL-C), globulin, albumin-to-globulin ratio (ALB/GLO), AST/ALT ratio, and urea levels (n = 6–8 mice per group) (b) Cerebellar tissue biochemical assays demonstrating that UA-36 restored mitochondrial function and redox balance, as indicated by increased ATP levels and mitochondrial DNA (mtDNA) copy number, alongside reduced reactive oxygen species (ROS) levels. Notably, hydrogen peroxide (H₂O₂) and nitric oxide (NO) levels were modulated following treatment. UA-36 also rebalanced pro- and anti-inflammatory cytokines, specifically modulating TNF-α, IL-4, and IL-10. Relative mRNA expression levels of human frataxin (hFXN) and p62 are also shown (n = 6–8 mice per group). All data in (a-b) are presented as mean ± SEM (n = 6–8 mice per group). Statistical significance was determined by one-way ANOVA with Tukey's post-hoc test. *p < 0.05, **p < 0.01, ***p < 0.001, ****p < 0.0001; ns, not significant. MDA, malondialdehyde; TG, triglycerides; HDL-C, high-density lipoprotein cholesterol; AST, aspartate aminotransferase; ALT, alanine aminotransferase; ALB, albumin; GLO, globulin; ATP, adenosine triphosphate; mtDNA, mitochondrial DNA; ROS, reactive oxygen species; H₂O₂, hydrogen peroxide; NO, nitric oxide; TNF-α, tumor necrosis factor-alpha; IL, interleukin; hFXN, human frataxin.


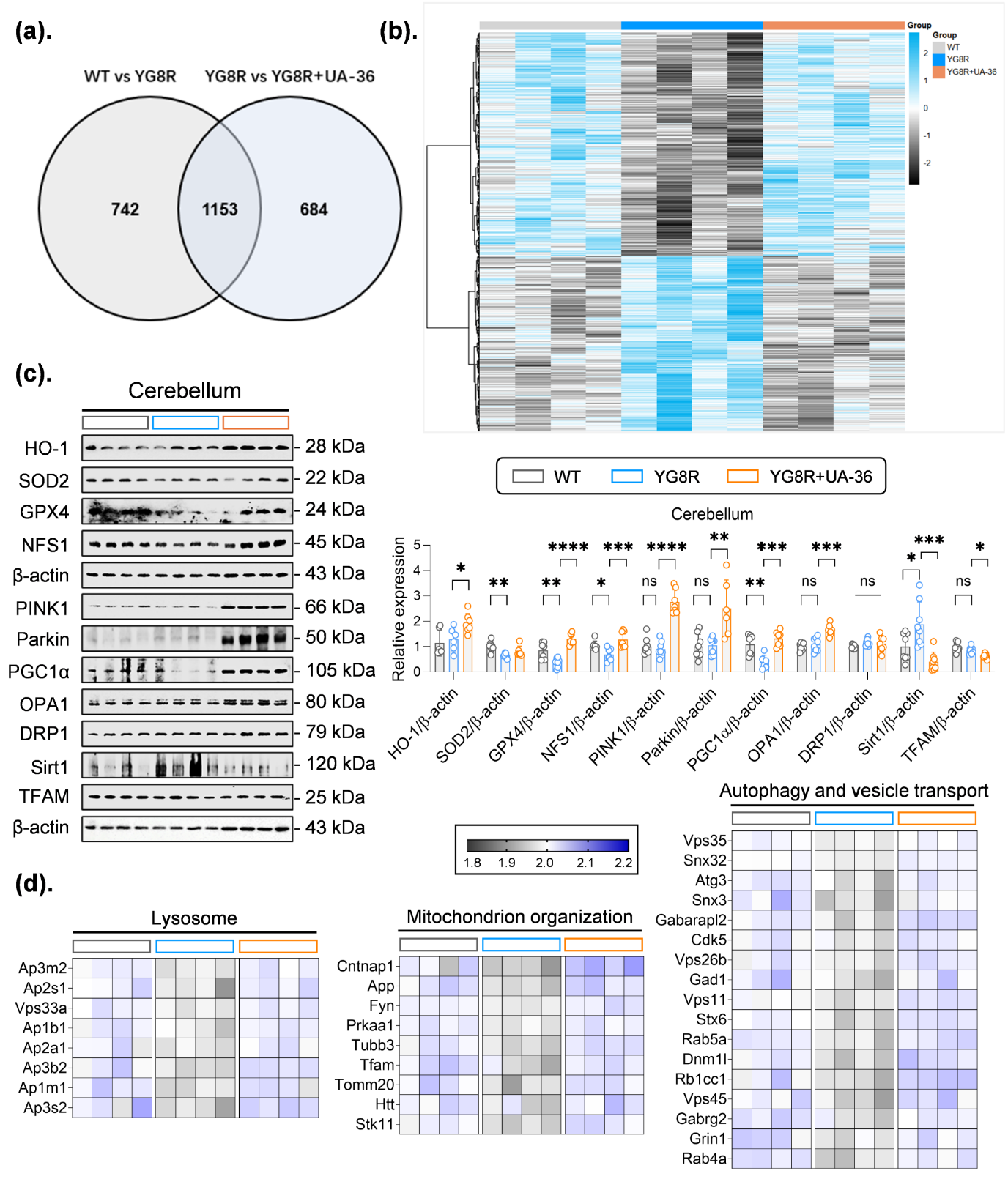


**Fig. S3** UA-36 restores mitochondrial proteostasis and lysosomal organization in vivo (a) Venn diagram showing the overlap of differentially expressed proteins identified by quantitative proteomics. Comparisons are shown for WT versus YG8R (742 upregulated, 1,153 downregulated) and YG8R versus YG8R+UA-36 (684 proteins altered), illustrating the partial rescue of the YG8R proteome by UA-36 treatment (b) Heatmap summarizing the expression patterns of proteins involved in autophagy and vesicle transport across experimental groups, demonstrating the coordinated dysregulation in YG8R mice and its partial normalization following UA-36 treatment (c) Representative immunoblots validating the expression of key proteins involved in antioxidant defense (HO-1, SOD2, GPX4), iron-sulfur cluster biogenesis (NFS1), mitophagy (PINK1, Parkin), mitochondrial biogenesis (PGC-1α, TFAM), mitochondrial dynamics (OPA1, DRP1), and metabolic regulation (Sirt1) in the cerebellum of WT, YG8R, and YG8R+UA-36 mice. β-actin served as a loading control. Molecular weights are indicated (d) Heatmaps showing the coordinated regulation of proteins involved in lysosomal function and mitochondrial organization, indicating pathway-level rescue by UA-36. Proteomic data in (a), (b), and (d) are derived from n = 4 mice per group. Immunoblot validation in (c) used n = 4–6 mice per group. HO-1, heme oxygenase-1; SOD2, superoxide dismutase 2; GPX4, glutathione peroxidase 4; NFS1, cysteine desulfurase; PINK1, PTEN-induced kinase 1; PGC-1α, peroxisome proliferator-activated receptor gamma coactivator 1-alpha; OPA1, optic atrophy 1; DRP1, dynamin-related protein 1; Sirt1, sirtuin 1; TFAM, mitochondrial transcription factor A.


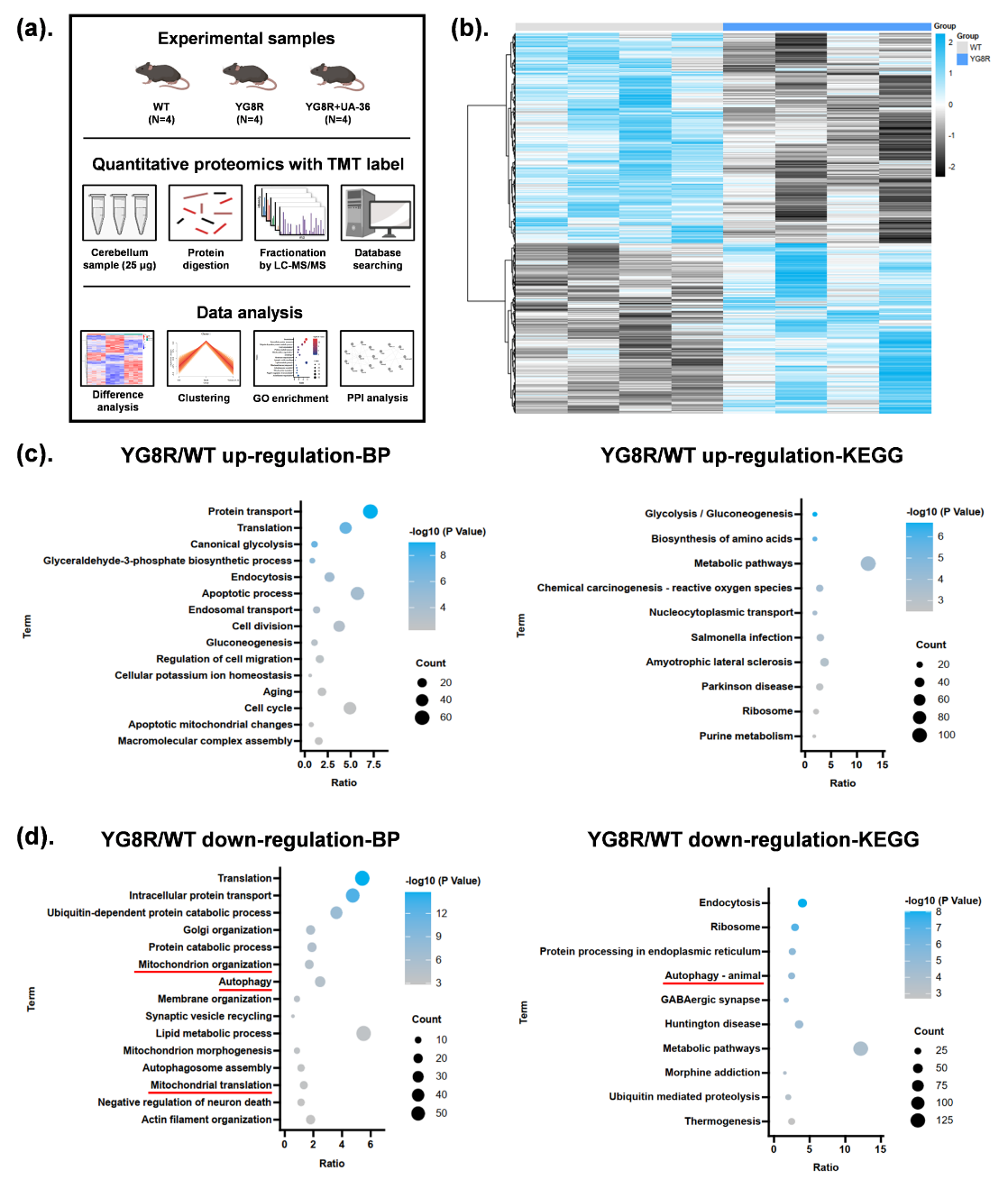


**Fig. S4** Quantitative proteomic workflow and pathway enrichment analysis in the cerebellum of YG8R mice (a) Schematic representation of the proteomic pipeline, including sample collection from wild-type (WT), YG8R, and UA-36-treated YG8R mice (n = 4 per group), protein extraction from cerebellum tissue, TMT-labeling, LC-MS/MS fractionation, and subsequent bioinformatic data analysis (differential expression, clustering, GO enrichment, and PPI analysis) (b) Hierarchical clustering heatmap showing the differential protein expression profiles comparing WT (gray) and YG8R (blue) groups. Rows represent individual proteins, and columns represent biological replicates. Color intensity indicates the log-transformed fold change (blue: upregulation; gray/black: downregulation) (c) Gene Ontology (GO) Biological Process (BP, left) and KEGG pathway analysis (right) for proteins significantly upregulated in YG8R compared to WT mice. Top enriched terms include protein transport, translation, and metabolic pathways such as glycolysis and amino acid biosynthesis (d) GO BP (left) and KEGG pathway analysis (right) for proteins significantly downregulated in YG8R mice. Notable enriched terms include translation, intracellular protein transport, and autophagy (underlined in red), suggesting a deficit in cellular degradation and mitochondrial organization in the YG8R model. For both (c) and (d), the x-axis represents the enrichment ratio; bubble size represents protein count; and color gradient represents the statistical significance (-log10 P-value).

**Procedures for the synthesis of UA-36**

The reference for the synthesis method of compound **UAS03**. DOI: 10.1038/s41467-018-07859-7.

To a solution of 4-methylpiperazine-1-formyl chloride hydrochloride (500 mg, 2.5 mmol) in CH_3_CN (20 mL) was added **UAS03** (250 mg, 1.2 mmol) and potassium carbonate (453 mg, 3.28 mmol) in order, and the mixture was stirred at 80℃ for 6 h. Then the mixture was extracted with ethyl acetate, dried over Na_2_SO_4_ and filtered. The filtrate was concentrated and purified by silica gel chromatography (DCM/MeOH = 50:1) to give **Compound I** (492 mg, 88%) as a white solid.

**^1^H NMR (400 MHz, CDCl_3_)** δ 7.57 (t, *J* = 8.6 Hz, 2H), 7.03 (dd, *J* = 8.5, 2.5 Hz, 1H), 6.87 (d, *J* = 2.4 Hz, 1H), 6.75 (dd, *J* = 8.4, 2.4 Hz, 1H), 6.70 (d, *J* = 2.3 Hz, 1H), 5.02 (s, 2H), 3.59 (br, 8H), 2.41 (t, *J* = 5.1 Hz, 8H), 2.29 (s, 6H). **^13^C NMR (101 MHz, CDCl_3_)** δ 155.18, 153.57, 153.45, 152.06, 150.82, 132.18, 127.14, 123.77, 123.07, 121.80, 119.97, 118.20, 115.83, 111.09, 68.45, 54.84, 54.84, 54.68, 54.68, 46.26, 46.26, 44.53, 44.53, 43.97, 43.97. ESI-MS m/z calcd for C_25_H_30_N_4_O_5_ [M + H] +: 467.22, found 467.3. HPLC purity: 100.0% (λ = 254 nm).

To a solution of **Compound, I** (93 mg, 0.2 mmol) in carbinol (10 mL) was added solution of ethyl acetate hydrochloride (4 mol/L, 1 mL), and was stirred at room temperature for 5 h. Then the mixture was filtered and collected the filter residue to give the white solid compound **UA-36** (98 mg, 92%) as a white solid.

**^1^H NMR (400 MHz, D_2_O)** δ 7.78– 7.70 (m, 2H), 7.11 (dd, *J* = 8.5, 2.4 Hz, 1H), 6.97 (d, *J* = 2.4 Hz, 1H), 6.84 (dd, *J* = 8.5, 2.4 Hz, 1H), 6.76 (d, *J* = 2.4 Hz, 1H), 5.07 (s, 2H), 4.48 – 4.33 (m, 2H), 4.28 – 4.17 (m, 2H), 3.59 – 3.50 (m, 4H), 3.48 – 3.39 (m, 2H), 3.36 – 3.25 (m, 2H), 3.21 – 3.10 (m, 4H), 2.91 (s, 6H). ESI-MS m/z calcd for C_25_H_30_N_4_O_5_ [M + H] +: 467.22, found 467.3. HPLC purity: 100.0% (λ = 254 nm).

**Identification for Compound I and UA-36**


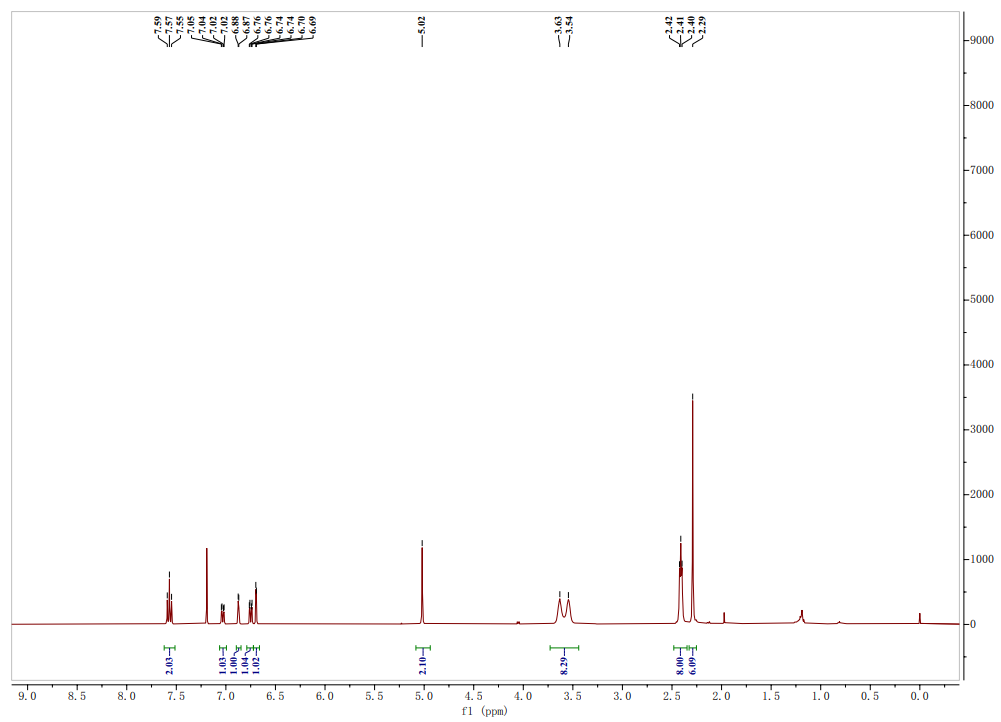


**NMR hydrogen spectrum results of Compound I**


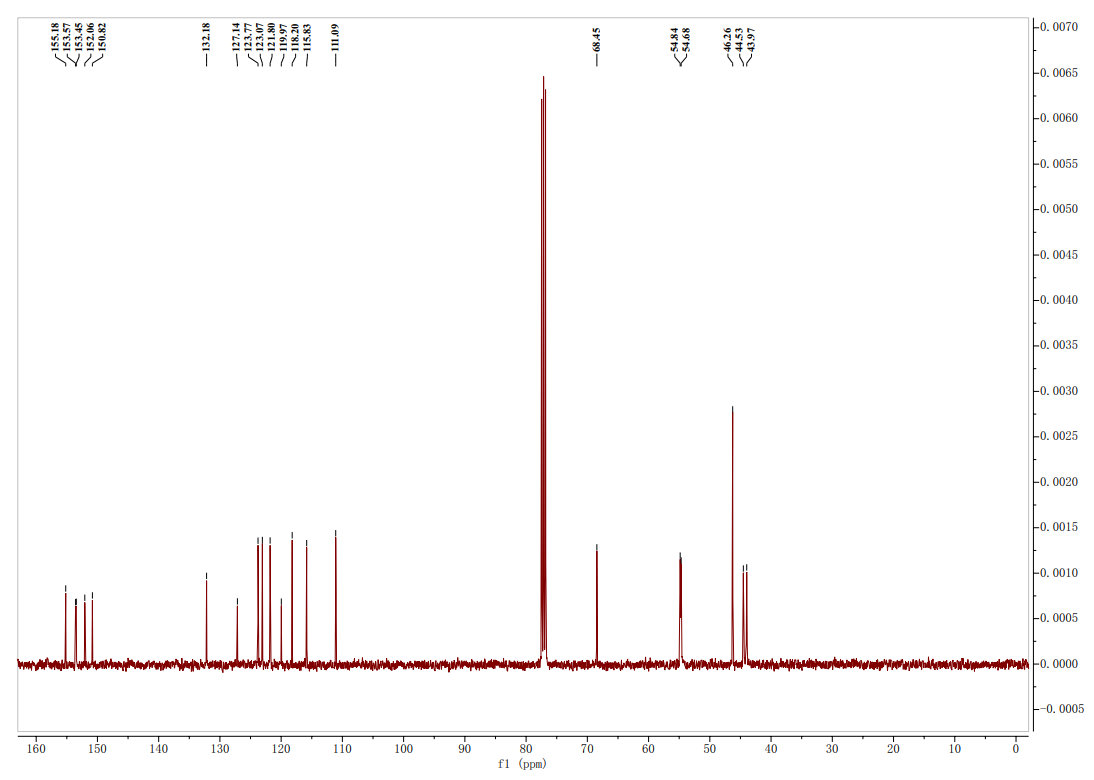


**NMR carbon spectrum results of Compound I**


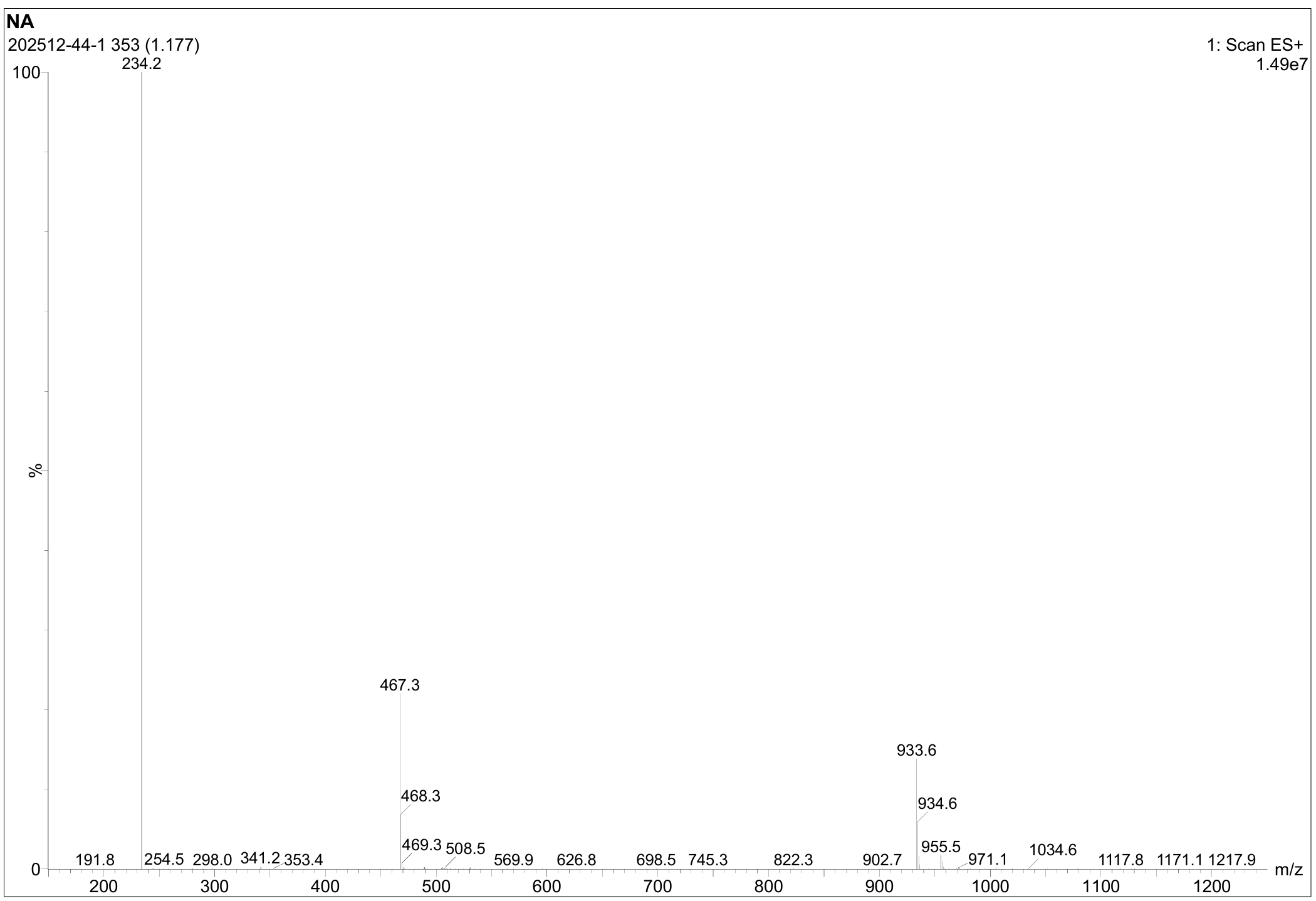


**Mass spectrum result of Compound I**


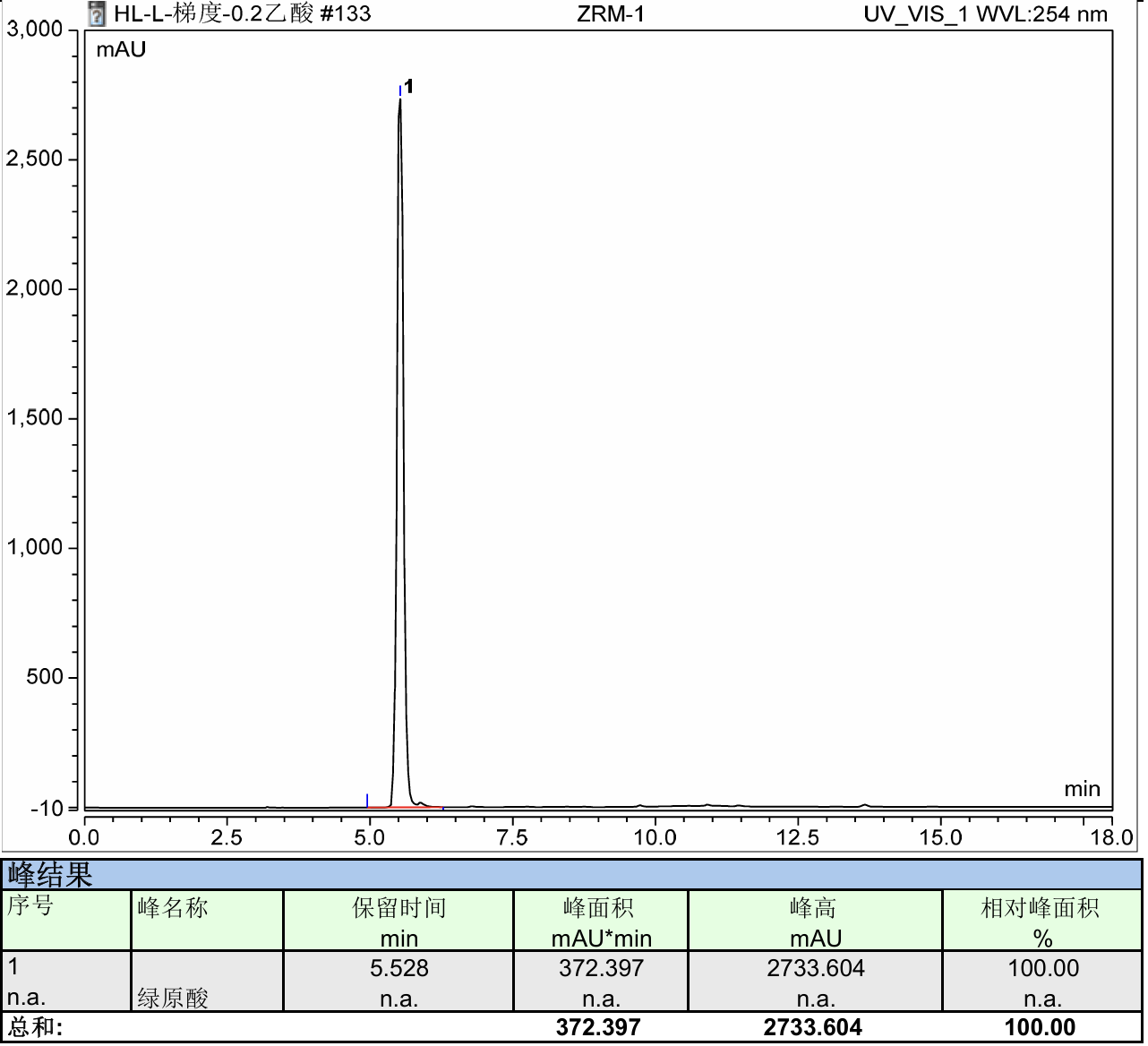


| No. | Ret. Time  min | Area  mAU*min | Height  mAU | Area  % |
| --- | --- | --- | --- | --- |
| 1 | 5.528 | 372.397 | 2733.604 | 100.00 |

**Liquid chromatography result of Compound I**


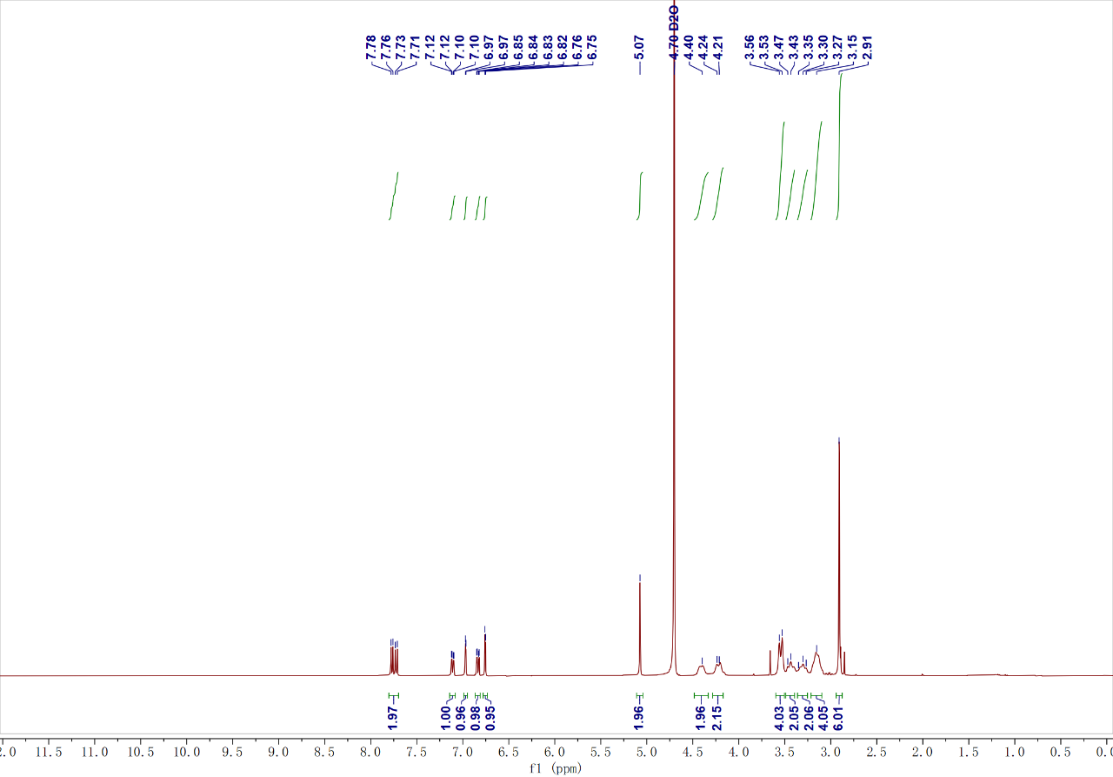


**NMR hydrogen spectrum results of UA-36**


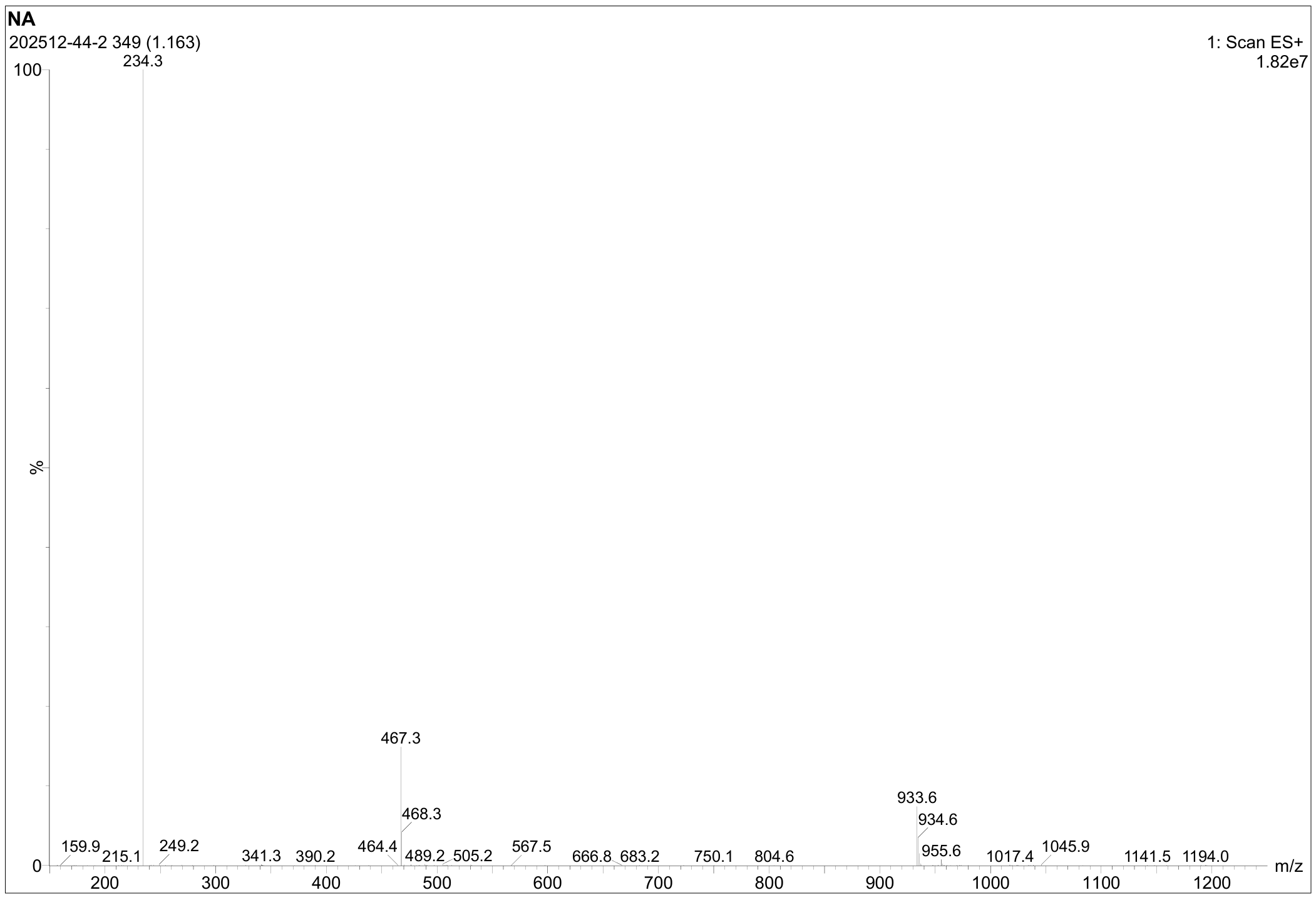


**Mass spectrum result of UA-36**


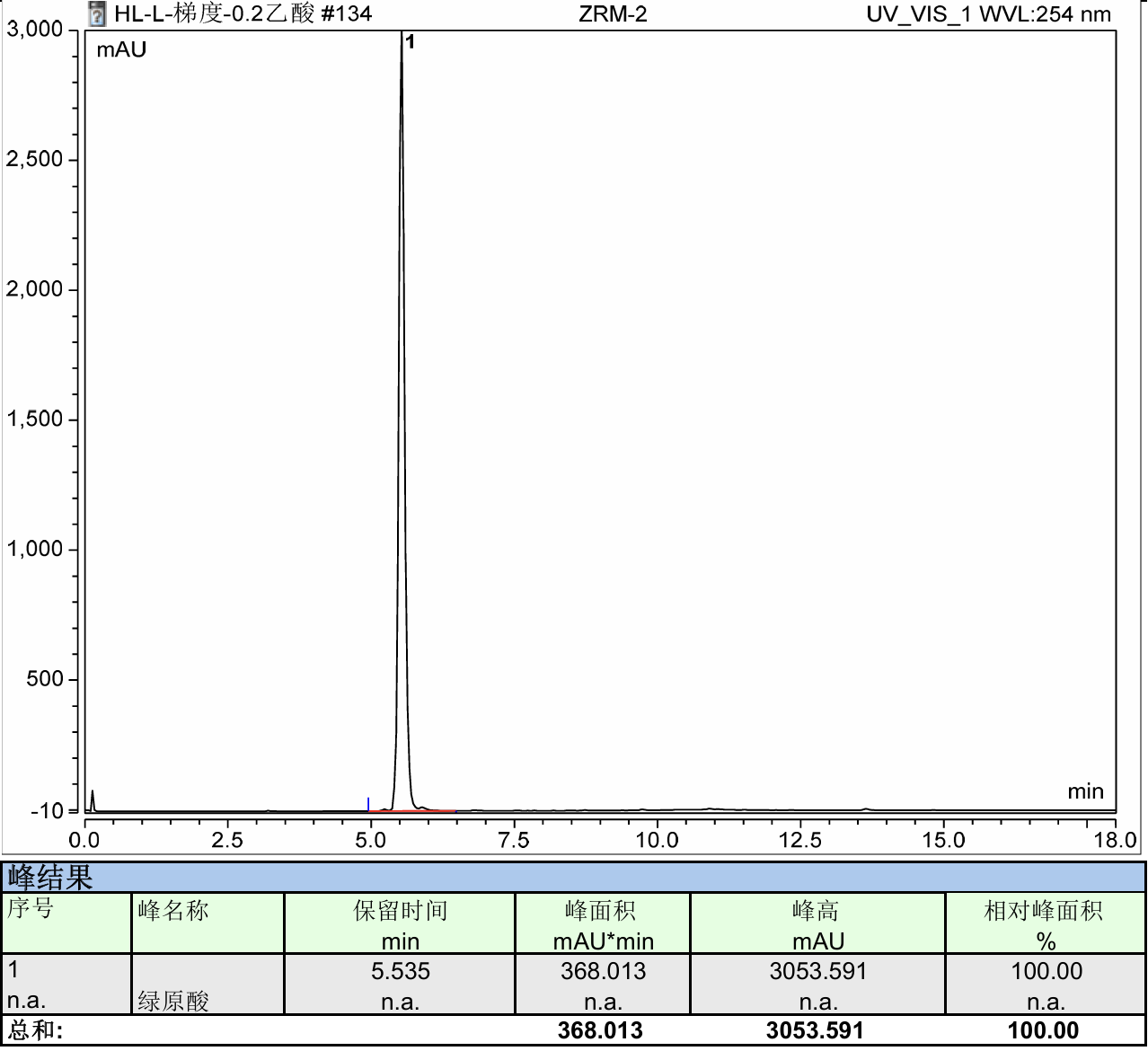


| No. | Ret. Time  min | Area  mAU*min | Height  mAU | Area  % |
| --- | --- | --- | --- | --- |
| 1 | 5.535 | 368.013 | 3053.591 | 100.00 |

Column: XBridge^®^ C18 3.5μm，4.6×50mm

Column Temp: 30 ℃

Mobile Phase A: 0.5% HCOOH in water

Mobile Phase B: MeOH

Flow Rate: 1.0ml/min

**Liquid chromatography result of UA-36**

**Supplementary materials and methods**

**Behaviors Analysis**

**Pole Test**

A custom-made wooden pole (50 cm height, 1 cm diameter) was vertically fixed to a stable base and wrapped with medical-grade gauze to prevent slipping while ensuring consistent grip across trials.

Mice were acclimatized to the testing room for ≥30 minutes before trials to reduce stress. Prior to testing, each mouse underwent two training sessions (one session/day for two consecutive days) to familiarize them with descending the pole. During training, mice were gently placed at the top of the pole, and guidance was provided if they failed to descend within 60 seconds. During the test, mice were placed head-up at the top of the pole, and the time to fully descend (all four paws touching the base) was recorded. Each mouse performed three trials with a 5-minute rest interval between trials to avoid fatigue. A maximum cutoff time of 120 seconds was enforced; if a mouse failed to descend, the trial was terminated, and the latency was recorded as 120 seconds.

**Beam Walk Test**

Balance, gait, and motor coordination were assessed using a beam walk test adapted from established protocols (REF). A 100 cm long, 12-mm wide wooden beam, painted white and segmented into 10 cm intervals, was horizontally positioned 20 cm above a foam-padded surface. Mice were acclimated to the testing room for at least 30 minutes under dim lighting. One day prior to testing, mice underwent two training trials on a wider (30 mm) beam, with a dark escape box (15 × 15 cm) placed at the far end to encourage traversal. During testing, mice were placed at one end of the 12-mm beam, and the time to reach the escape box was recorded. Three trials were performed per mouse, with 5-minute rest intervals between trials. A 60-second cutoff time was applied; mice failing to cross within this limit were gently guided to the escape box, and a latency of 60 seconds was assigned.The average traversal time across the three trials was used for analysis.

**Grip Strength Test**

A mouse grip tester (YLS-13A, Nuolei Xinda Technology Co., LTD, China) was used to quantitatively evaluate the grip strength of mice. Mice were acclimated to the apparatus for 5 minutes before testing. During testing, mice were gently lowered by the base of the tail until they grasped the grid with both forepaws. They were then pulled horizontally at a consistent speed and approximately 15° angle until grip release, with peak force (Newtons) recorded. Five consecutive trials, separated by 1-minute rest intervals, were performed to mitigate fatigue. The maximum recorded force was used for analysis, with values exceeding 20% deviation from the mean excluded as outliers. All tests were conducted at the same time of day to minimize circadian rhythm effects.

**Wire Hanging Test**

The wire-hanging test evaluates forelimb grip strength and endurance in mice. Each mouse was gently placed on a horizontal wire mesh grid (20 × 20 cm, 2 mm diameter) and allowed to grip firmly with its forepaws. The grid was then slowly inverted and elevated to a height of 50 cm above a soft foam-padded surface (10 cm thick) to prevent injury upon falling.

Each mouse underwent three trials with a minimum 30-minute rest interval between trials to avoid fatigue. The latency to fall was recorded with a maximum cutoff time of 180 seconds per trial. Mice were closely monitored for signs of distress, and trials were immediately terminated if any stress-related behaviors (e.g., vocalization, excessive struggling) were observed.

**Gait Analysis**

Quantitative gait assessment was performed using a computerized automated gait analysis system (BT60601, Stones Scientific Instruments, China) with the following specifications and protocol: Two synchronized high-speed digital cameras (100 fps) orthogonally positioned and illuminated with infrared LEDs (850 nm) for dark environment recording. A calibrated walking runway (50 × 5 × 1 cm) with adjustable plexiglass walls was used. Mice were acclimated to the testing room for 30 minutes under dim red light and underwent three training trials on the runway. During testing, mice traversed the darkened runway, and at least five complete stride cycles (three consecutive steps) were captured per trial. Three successful trials were obtained per mouse, with 5-minute inter-trial rest periods. The following gait parameters were analyzed: spatial parameters (stride length, base of support, paw angle), temporal parameters (stance phase duration, swing speed, step cycle frequency), paw pressure distribution, and interlimb coordination. The runway was cleaned with 75% ethanol between animals, and fresh paper lining was replaced after every fifth animal. Videos were analyzed using manufacturer software (v2.1) with manual verification. Outliers (>2 SD from the group mean) were excluded after blinded analysis. Parameters were normalized to body length for inter-animal comparison. System calibration was verified daily using standard reference objects. The ambient temperature was maintained at 22 ± 1°C throughout testing.

**Staining Procedures**

Mice were deeply anesthetized with sodium pentobarbital (50 mg/kg, i.p.) until the absence of a pedal reflex. Perfusion was performed with 0.1 M phosphate-buffered saline (PBS, pH 7.4, 4°C) at 10 mL/min for 5 minutes, followed by 4% paraformaldehyde (PFA) in 0.1 M PBS (4°C) for 10 minutes. Target tissues (cerebellum, spinal cord [specify segment], heart [specify ventricle/region], and gastrocnemius muscle) were rapidly dissected, trimmed to 5×5×3 mm blocks, and immersion-fixed in 4% PFA at 4°C for 24 hours (neural tissues) or 48 hours (muscular/cardiac tissues). Samples were then dehydrated through a graded ethanol series (70%, 80%, 95%, 100% ×2; 1 hour each), cleared in xylene (3 changes, 1 hour each), and paraffin-embedded (58-60°C; 3 changes, 1 hour each). Serial sections (5 μm) were cut using a rotary microtome (RM2235, Leica, Germany), mounted on poly-L-lysine coated slides, and dried overnight at 37°C.

**Hematoxylin and Eosin (H&E):** Cerebellum, heart, and gastrocnemius muscle sections were dewaxed in xylene and rehydrated to water through a graded ethanol series. Sections were stained with hematoxylin (10 minutes), washed in distilled water, differentiated, stained with eosin (2 minutes), dehydrated through a graded ethanol series, cleared in xylene, and mounted with neutral gum.

**Nissl:** Cerebellum and L4-L5 spinal cord sections were dewaxed and rehydrated as above. Sections were stained with Nissl staining solution (45 minutes at 60°C), washed in distilled water, dehydrated through a graded ethanol series, cleared in xylene, and mounted with neutral gum.

**Prussian Blue Iron:** As described earlier, cerebellum sections were dewaxed and rehydrated. Sections were stained using a Prussian Blue Iron Stain Kit (G1428, Solarbio, China) according to the manufacturer's instructions: Perls solution (20 minutes at 37°C), distilled water wash, working solution, and enhancement solution (10-20 minutes), distilled water wash, graded ethanol dehydration, xylene clearing, and mounting with neutral gum.

**Sirius red staining:** Muscle sections were stained using a Modified Sirius Red Stain Kit (G1472, Solarbio, China) according to the manufacturer's instructions. sections were stained with Sirius red solution for 10 minutes, briefly rinsed with distilled water to remove excess dye, dehydrated through a graded ethanol series, cleared in xylene, and mounted with neutral gum.

**Image Acquisition and Analysis:** Every 10th section was stained with H&E for quality control. As specified, selected sections underwent Nissl, Masson's trichrome, or immunohistochemical staining. Digital images were acquired using an Olympus VS200 slide scanner with a 20× objective. Quantitative analysis was performed using ImageJ with custom macros.

**Enzyme-Linked Immunosorbent Assay (ELISA)**

The frozen tissue samples were ultrasonically homogenized with cold RIPA buffer (P0013B, Beyotime, China) containing protease inhibitors (20124ES, Yisheng Biotechnology, China) and centrifuge at 12,000 ×g at 4°C for 15 min. The protein concentration was determined by BCA assay (P0011, Beyotime, China). ELISA kit according to the instructions on using the reagents provided experiment (RK00008/RK00027/RK00036/RK00016/RK04770, Abclonal, China). We use a 96-well plate pre-packaged with the captured antibody, which is successfully diluted with the corresponding standard product to produce a six-point standard curve. The remaining 20 µL sample and the corresponding volume of diluent were added to each well as a negative control. Next, the board was incubated at 37℃ for 2 hours. After incubation, the liquid in the hole was discarded and each hole was washed three times with the Wash Buffer provided by the kit. Then 100µL biotin was added to detect the antibody and incubated at 37℃ for 1 hour. The liquid in the hole was discarded and washed three times, and a streptavidin-HRP working solution was added at room temperature for 30 minutes. Next, the TMB substrate solution is added, and after 15 minutes of reaction in a dark place, the reaction is stopped with a termination solution. The light absorption value of the liquid at 450nm in each well was measured using an enzyme labeler within 15 minutes. The concentration of the measured index in each hole was calculated according to the standard curve, and the value was normalized to the protein concentration for statistics.

**Proteomics**

**Sample Preparation**

Take cerebellar tissues from each group of animals (n=4) and place them in PBS solutions containing 8M urea, protease, and phosphatase inhibitors. Then, lyse the tissues using low-temperature ultrasound (VCX-150, Sonics, USA). After the sample was lysed and centrifuged at 4 ℃ and 12000 × g for half an hour, the upper clear liquid was taken for BCA protein quantification (P0011, Beyotime, China). Take 25 μg of protein from each sample and add dithiothreitol (DTT, final concentration 10 mM). Mix well and incubate at 55 ℃ for 1 hour; Incubate with iodoacetamide (IAA, final concentration 25 mM) at room temperature and in dark conditions for 1 hour.

Then, pancreatic enzyme (1:25w/w) (V5072, Promega, USA) was added to the sample and digested at 37 ° C for 4 hours. Then, dilute the urea concentration in the sample to 1M using PBS (pH=8.0) and continue to digest at 37 °C for 11 hours. After digestion, adjust the pH of the solution to 1-2 with trifluoroacetic acid (TFA), and desalinate it in a desalination column (OASIS HLB, USA) before drying at low temperature.

**TMT Labeling and LC-MS/MS Analysis**

The dried sample was re-dissolved in 50 μL 200mM triethylammonium bicarbonate (TEAB) and then labeled with TMT reagent at room temperature for 1 hour. The reaction was terminated with 5% hydroxylamine. Mix samples with different labels, desalinate them through a desalination column, dry them at low temperature, and dissolve them again in 100 μL of 0.1% formic acid. Add the labeled peptide to the chromatography column (WAT094225, Waters, USA) and separate the peptide sample into 60 components using Extreme 3000 UHPLC (Thermo Fisher Science, USA). All components were combined into 15 tubes by centrifugation and low-temperature drying and dissolved in 30 μ L of 0.1% formic acid. After vortex centrifugation, the upper clear liquid is taken and passed through Liquid chromatography (LC) mass spectrometry (MS)/MS analysis.

Finally, proteomics discovery software was used to calculate the relative expression levels of each protein in the mouse database based on the reported ion strength of each peptide. Using the t-test method in Perseus software, the differential expression of proteins in the cerebellar tissues of mice in the model group and control group, as well as the model group and UA-36 treatment group, was set to a p-value less than 0.05.

**Western Blot**

Cerebellar tissue and cell samples were ultrasonically lysed in RIPA buffer containing protease inhibitors, supernatant was collected after centrifugation, and BCA protein quantification was performed as described above. The supernatant is mixed with the sample buffer and boiled at 100°C for 10 minutes for denaturation. The SDS-PAGE gel was prepared in advance, the boiled protein samples were added, and the electrophoresis system (Bio-Rad, USA) was used for separation. After the protein was separated by molecular weight, the protein was horizontally transferred to the nitrate cellulose membrane (66485, Pall, USA) by electrophoresis. At room temperature, the membrane was closed with TBST (tris buffered brine, 0.1% Tween 20) containing 5% skim milk powder for 1 hour to prevent non-specific binding. After closure, the membrane was washed three times with TBST, cut according to the molecular weight of the desired target protein, and incubated at 4℃ overnight. The next day, the primary antibody was recovered, the membrane was cleaned with TBST, and the secondary antibody (anti-rabbit or anti-mouse, depending on the primary antibody) coupled with horseradish peroxidase (HRP) was incubated. The protein bands were imaged with the Bio-Rad ChemiDoc XRS+ imaging system (Bio-Rad Laboratories) after TBST was washed several times. Density analysis of protein bands was performed using ImageJ software (National Institutes of Health, Bethesda, USA). Band strength was normalized to load control (beta-actin) for total protein analysis or corresponding total protein levels for phosphorylated protein analysis.

**Quantitative real-time PCR**

Total RNA was isolated from cerebellar tissue using TRIzol reagent (RC112, Vazyme, China). cDNA was then generated with the HiScript IV All-in-One Ultra RT SuperMix Kit (R433, Vazyme, China). qRT-PCR was carried out using SupRealQ Ultra Hunter SYBR qPCR Master Mix (Q713, Vazyme, China) in accordance with the manufacturer protocol, with each sample analyzed in at least duplicate. The relative transcript levels were normalized to β-actin expression. The primary information is as follows.

Primer sequences for p62.

(F) GGGAACACAGCAAGCTCATC

(R) TGTCAACCTCAATGCCTAGAG.

Primer sequences for β-actin.

(F) ACCAGAGGCATACAGGGACA

(R) CTAAGGCCAACCGTGAAAAG

**Supplementary tables**

**Supplementary Table 1. Changes of drug concentration in the plasma after a single oral gavage of UA-36(118.3 mg/kg, 0.22 mol) in ICR mice. n=3.**

| Time (h) | Concentration of UA-36 (ng/mL) | | | Mean | SD |
| --- | --- | --- | --- | --- | --- |
|  | No.1 | No.2 | No.3 |  |  |
| 0.25 | 625.18 | 1311.74 | 2038.17 | 1325.03 | 706.59 |
| 0.5 | 332.81 | 715.44 | 766.42 | 604.89 | 237.00 |
| 1 | 165.08 | 307.97 | 391.82 | 288.29 | 114.64 |
| 2 | 53.11 | 67.29 | 82.53 | 67.64 | 14.71 |
| 4 | 22.34 | 39.51 | 39.23 | 33.69 | 9.83 |
| 6 | 23.45 | 16.36 | 23.17 | 20.99 | 4.01 |
| 8 | 19.43 | 11.15 | 18.63 | 16.40 | 4.56 |
| 24 | BLQ | BLQ | BLQ | NA | NA |

**Supplementary Table 2. Changes of drug concentration in the plasma after a single oral gavage of UA (50 mg/kg, 0.22 mol) in ICR mice. n=3.**

| Time (h) | Concentration of UA (ng/mL) | | | Mean | SD |
| --- | --- | --- | --- | --- | --- |
|  | No.1 | No.2 | No.3 |  |  |
| 0.25 | 205.83 | 249.94 | 104.20 | 186.66 | 74.74 |
| 0.5 | 79.60 | 124.23 | 85.29 | 96.37 | 24.29 |
| 1 | 38.47 | 15.08 | 19.89 | 24.48 | 12.35 |
| 2 | 43.13 | 22.86 | 6.06 | 24.02 | 18.56 |
| 4 | 46.25 | 39.73 | 31.74 | 39.24 | 7.27 |
| 6 | 77.709 | 32.123 | 24.187 | 44.67 | 28.88 |
| 8 | 52.892 | 22.078 | 13.725 | 29.57 | 20.63 |
| 24 | 7.88 | BLQ | BLQ | NA | NA |

**Supplementary Table 3. Changes of PK parameter in the plasma after a single oral gavage of UA-36(118.3 mg/kg, 0.22 mol) in ICR mice. n=3.**

|  | No.1 | No.2 | No.3 | Mean | SD |
| --- | --- | --- | --- | --- | --- |
| T_1/2_ (h) | 1.99 | 2.21 | 2.78 | 2.33 | 0.4 |
| T_max_ (h) | 0.25 | 0.25 | 0.25 | 0.25 | 0 |
| C_max_ (ng/mL) | 625.18 | 1311.74 | 2038.17 | 1325.03 | 706.59 |
| AUC_(0-t)_ (h*ng/mL) | 595.58 | 1051.03 | 1358.03 | 1001.55 | 383.63 |
| AUC_(0-∞）_(h*ng/mL) | 651.47 | 1086.58 | 1432.67 | 1056.90 | 391.44 |
| MRT_(0-t)_ (h) | 1.68 | 1.19 | 1.14 | 1.34 | 0.30 |
| MRT_(0-∞)_ (h) | 2.47 | 1.52 | 1.71 | 1.90 | 0.50 |

**Supplementary Table 4. Changes of PK parameter in the plasma after a single oral gavage of UA (50 mg/kg, 0.22 mol) in ICR mice. n=3.**

|  | No.1 | No.2 | No.3 | Mean | SD |
| --- | --- | --- | --- | --- | --- |
| T_1/2_ (h) | 5.59 | 4.72 | 3.31 | 4.54 | 0.56 |
| T_max_ (h) | 0.25 | 0.25 | 0.25 | 0.25 | 0 |
| C_max_ (ng/mL) | 205.83 | 249.94 | 104.2 | 186.66 | 74.74 |
| AUC_(0-t)_ (h*ng/mL) | 961.83 | 320.45 | 207.61 | 496.63 | 406.81 |
| AUC_(0-∞）_(h*ng/mL) | 1025.37 | 470.76 | 273.1 | 589.74 | 390 |
| MRT_(0-t)_ (h) | 7.08 | 3.12 | 3.42 | 4.54 | 2.21 |
| MRT_(0-∞)_ (h) | 12.85 | 4.28 | 2.77 | 6.63 | 5.43 |

**Supplementary Table 5. List of antibodies.**

| Antibody | Company | Lot Number | Dilute | Source |
| --- | --- | --- | --- | --- |
| mFXN | Proteintech | 14147-1-AP | 1/1000 | Rabbit |
| Drp1 | Cell signaling technology | 8570 | 1/1000 | Rabbit |
| Mfn1 | Santa Cruz | sc-166644 | 1/500 | Mouse |
| OPA1 | Cell signaling technology | 80471 | 1/1000 | Rabbit |
| Beclin-1 | Cell signaling technology | 3738 | 1/1000 | Rabbit |
| LC3B | Abcam | ab48394 | 1/1000 | Rabbit |
| Nrf2 | Cell signaling technology | 12721 | 1/1000 | Rabbit |
| HO-1 | Cell signaling technology | 70081 | 1/1000 | Rabbit |
| NFS1 | Abcam | ab229829 | 1/1000 | Rabbit |
| p-AMPKα | Cell signaling technology | 2535 | 1/1000 | Rabbit |
| AMPKα | Cell signaling technology | 5832 | 1/1000 | Rabbit |
| PGC1α | Abcam | ab54481 | 1/1000 | Rabbit |
| NDUFA10 | Abcam | ab174829 | 1/1000 | Rabbit |
| SDHB | Abcam | ab14714 | 1/1000 | Mouse |
| UQCRFS1 | Abcam | ab14746 | 1/1000 | Mouse |
| COX5A | Abcam | ab110262 | 1/1000 | Mouse |
| ATP5A | Abcam | ab14748 | 1/1000 | Mouse |
| p-mTOR | Cell signaling technology | 2971 | 1/1000 | Rabbit |
| mTOR | Cell signaling technology | 2972 | 1/1000 | Rabbit |
| p-ULK1 | Cell signaling technology | 6888 | 1/1000 | Rabbit |
| ULK1 | Cell signaling technology | 8054 | 1/1000 | Rabbit |
| CTSD | Abcam | Ab75852 | 1/1000 | Rabbit |
| hFXN | Invitrogen | 45-6300 | 1/1000 | Mouse |
| Sirt1 | Cell signaling technology | 8469 | 1/1000 | Mouse |
| TFAM | Proteintech | 22586-1-AP | 1/1000 | Rabbit |
| LAMP1 | Abcam | ab25630 | 1/500 | Mouse |
| PINK1 | Abcam | ab23707 | 1/1000 | Rabbit |
| Parkin | Cell signaling technology | 4211S | 1/1000 | Mouse |
| ATG5 | Cell signaling technology | 12994 | 1/1000 | Rabbit |
| P62 | Cell signaling technology | 5114 | 1/1000 | Rabbit |
| NeuN | Sigma | MAB377 | 1/500 | Mouse |
| IBA1 | Cell signaling technology | 17198S | 1/1000 | Rabbit |
| GFAP | Cell signaling technology | 3670S | 1/1000 | Mouse |
| SOD2 | Cell signaling technology | 13194 | 1/1000 | Rabbit |
| GPX4 | Invitrogen | PA5-102521 | 1/1000 | Rabbit |
| Actin | Santa Cruz | sc-47778 | 1/500 | Mouse |
| mFXN | Proteintech | 14147-1-AP | 1/1000 | Rabbit |
| Drp1 | Cell signaling technology | 8570 | 1/1000 | Rabbit |
| Mfn1 | Santa Cruz | sc-166644 | 1/500 | Mouse |
