# Supplementary material for: Autophagy activation by urolithin-a derivative UA-36 mitigates Friedreich’s ataxia pathologies induced by frataxin deficiency
Source: Mol Biomed. 2026 Jun 4;7:82. doi: 10.1186/s43556-026-00457-w (PMC13237351; doi:10.1186/s43556-026-00457-w)
Supplement: Supplementary file 2 — Supplementary Material 2. [file 43556_2026_457_MOESM2_ESM.pdf]

Figure 1

N2a cell lysate

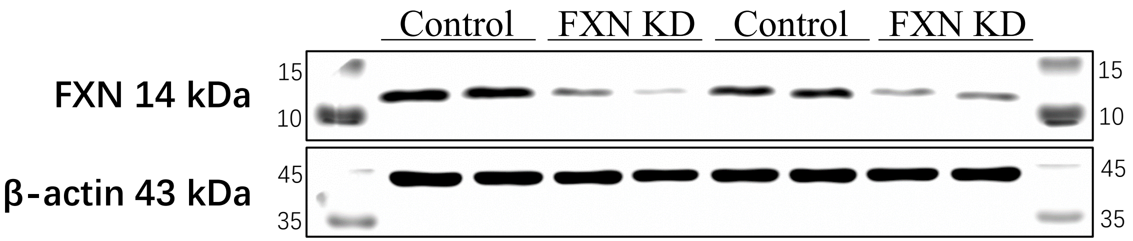

Figure 1

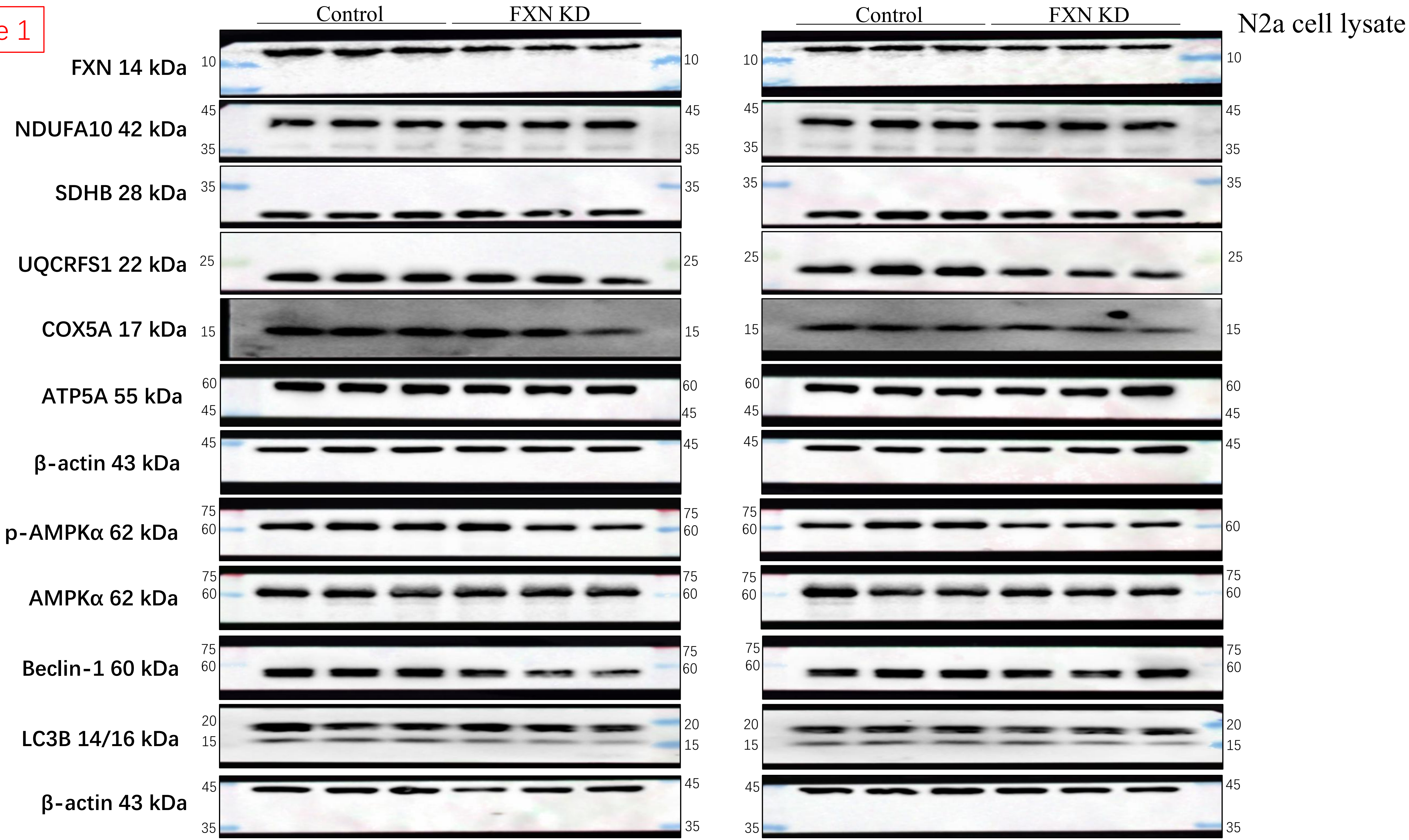
Control
FXN KD
N2a cell lysate
FXN 14 kDa
NDUFA10 42 kDa
SDHB 28 kDa
UQCRFS1 22 kDa
COX5A 17 kDa
ATP5A 55 kDa
β-actin 43 kDa
p-AMPKα 62 kDa
AMPKα 62 kDa
Beclin-1 60 kDa
LC3B 14/16 kDa
β-actin 43 kDa
10
45
35
25
15
60
75
20

Figure 2

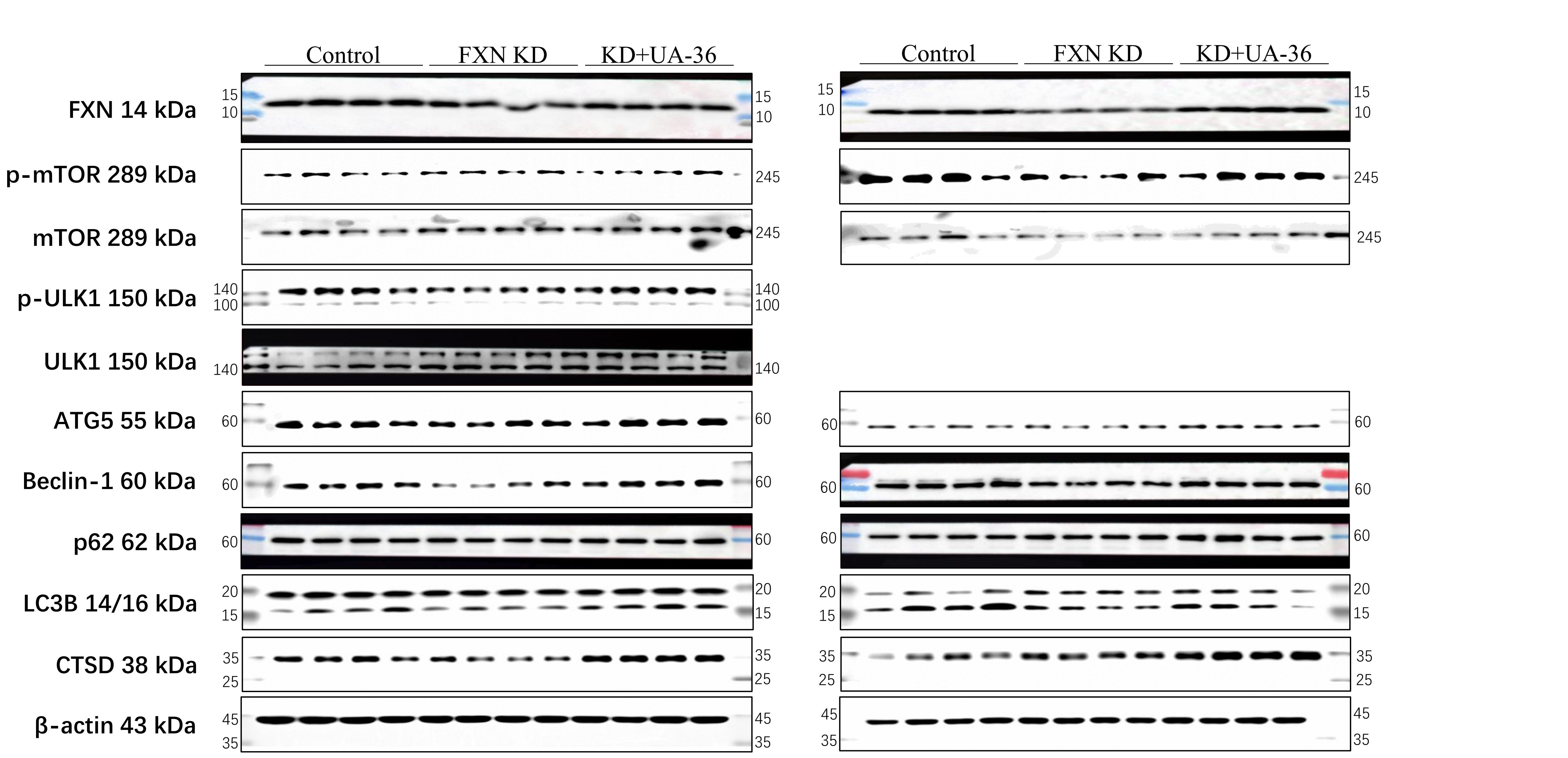
N2a cell lysate
Control
FXN KD
KD+UA-36
FXN 14 kDa
p-mTOR 289 kDa
mTOR 289 kDa
p-ULK1 150 kDa
ULK1 150 kDa
ATG5 55 kDa
Beclin-1 60 kDa
p62 62 kDa
LC3B 14/16 kDa
CTSD 38 kDa
β-actin 43 kDa
15
10
245
140
100
60
20
35
25
45

Figure 2

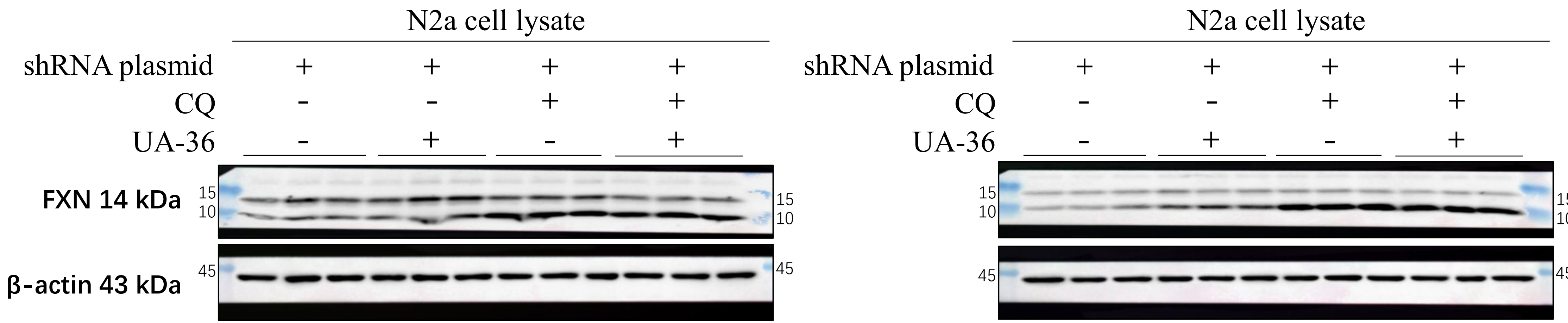
N2a cell lysate
shRNA plasmid + + + +
CQ - - + +
UA-36 - + - +
FXN 14 kDa
15
10
β-actin 43 kDa
45
N2a cell lysate
shRNA plasmid + + + +
CQ - - + +
UA-36 - + - +
15
10
45

Figure 2

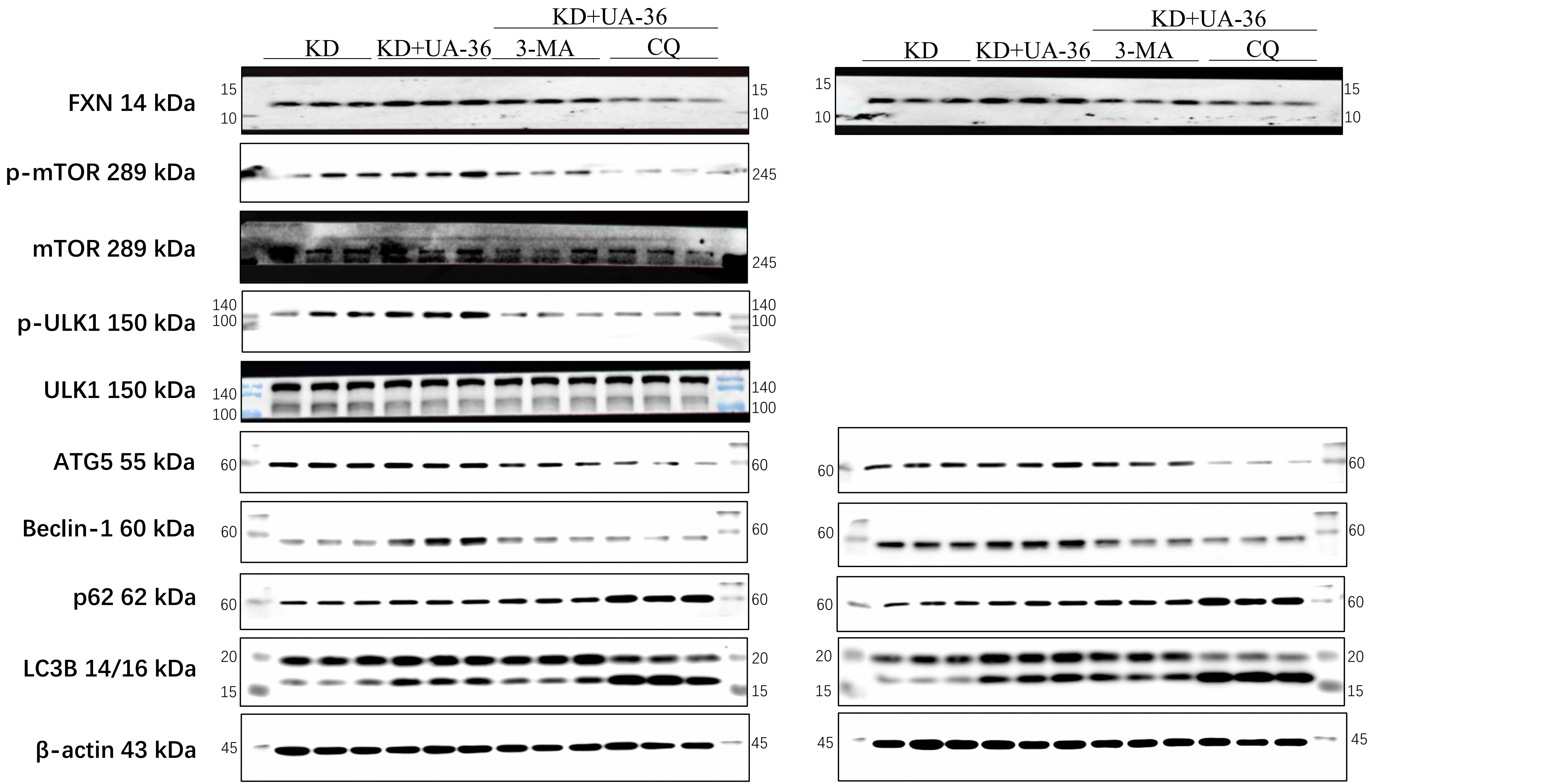
N2a cell lysate
KD+UA-36
KD
KD+UA-36
3-MA
CQ
FXN 14 kDa
p-mTOR 289 kDa
mTOR 289 kDa
p-ULK1 150 kDa
ULK1 150 kDa
ATG5 55 kDa
Beclin-1 60 kDa
p62 62 kDa
LC3B 14/16 kDa
β-actin 43 kDa
15
10
245
140
100
60
20
45

Figure 5

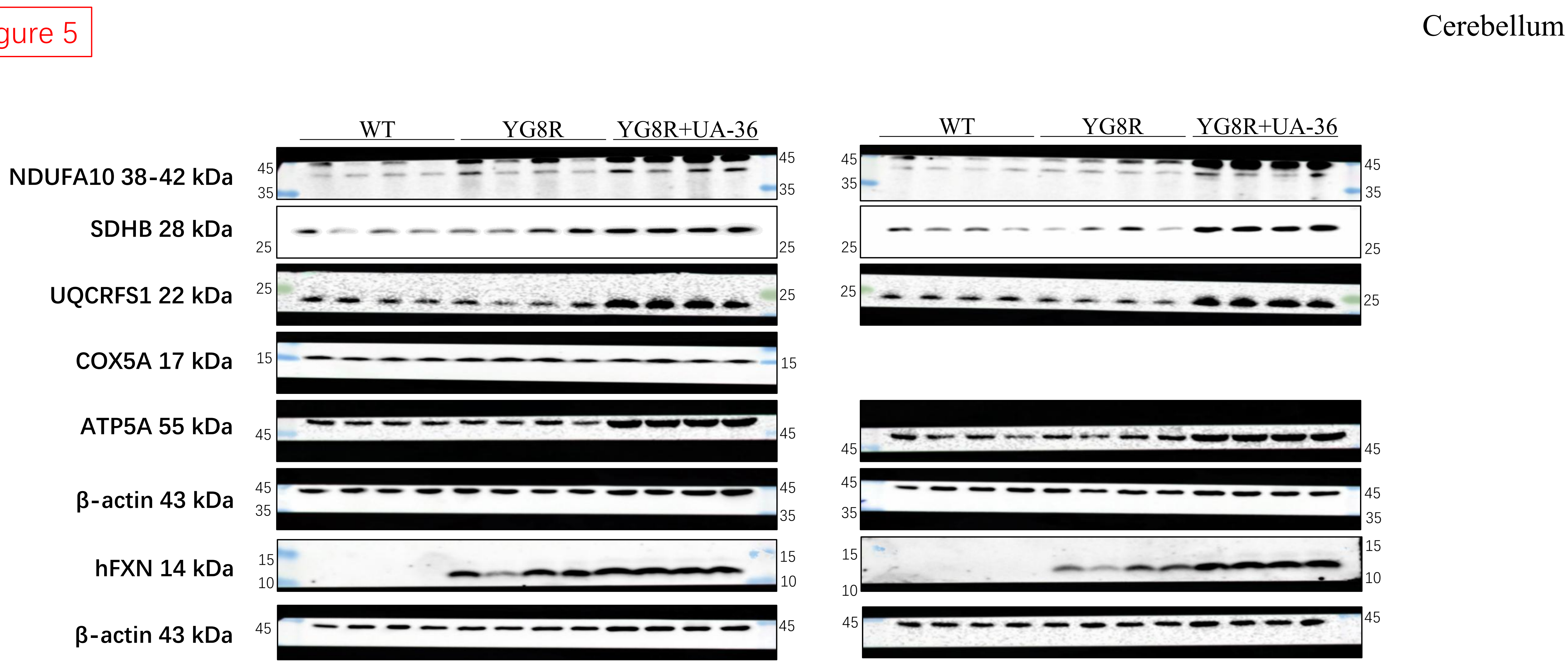
Cerebellum
WT
YG8R
YG8R+UA-36
NDUFA10 38-42 kDa
SDHB 28 kDa
UQCRFS1 22 kDa
COX5A 17 kDa
ATP5A 55 kDa
β-actin 43 kDa
hFXN 14 kDa
β-actin 43 kDa
45
35
25
15
10

Figure 5

Cerebellum

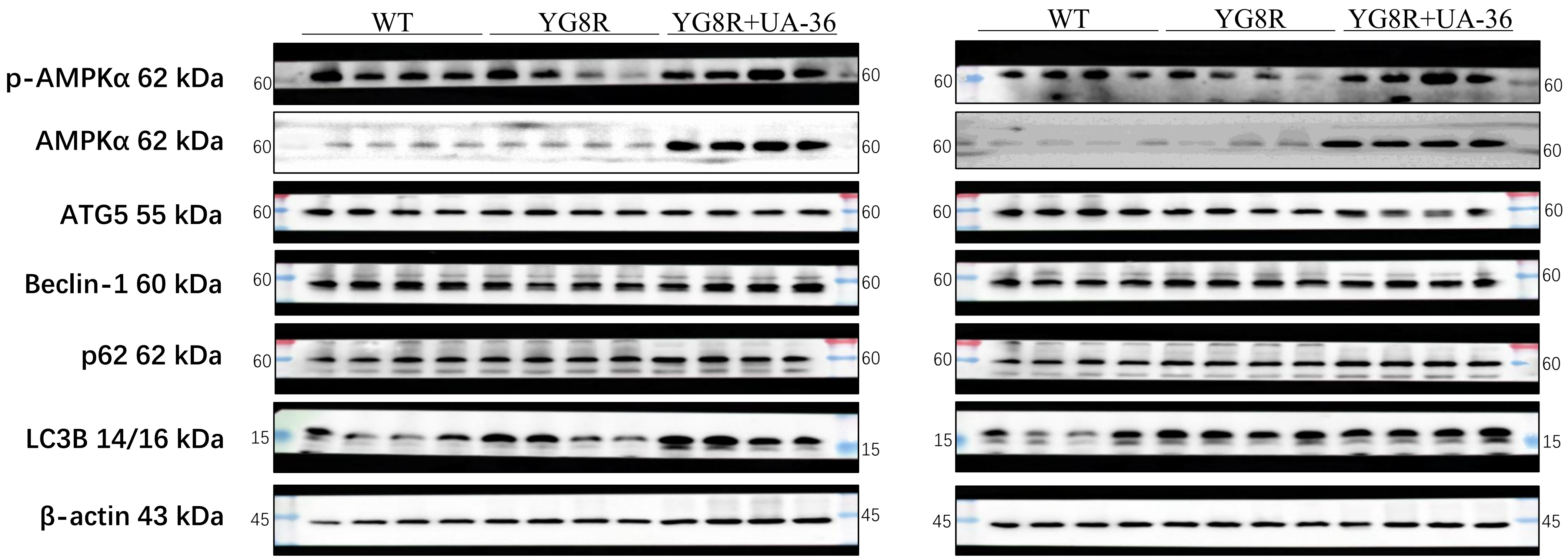

Figure S1

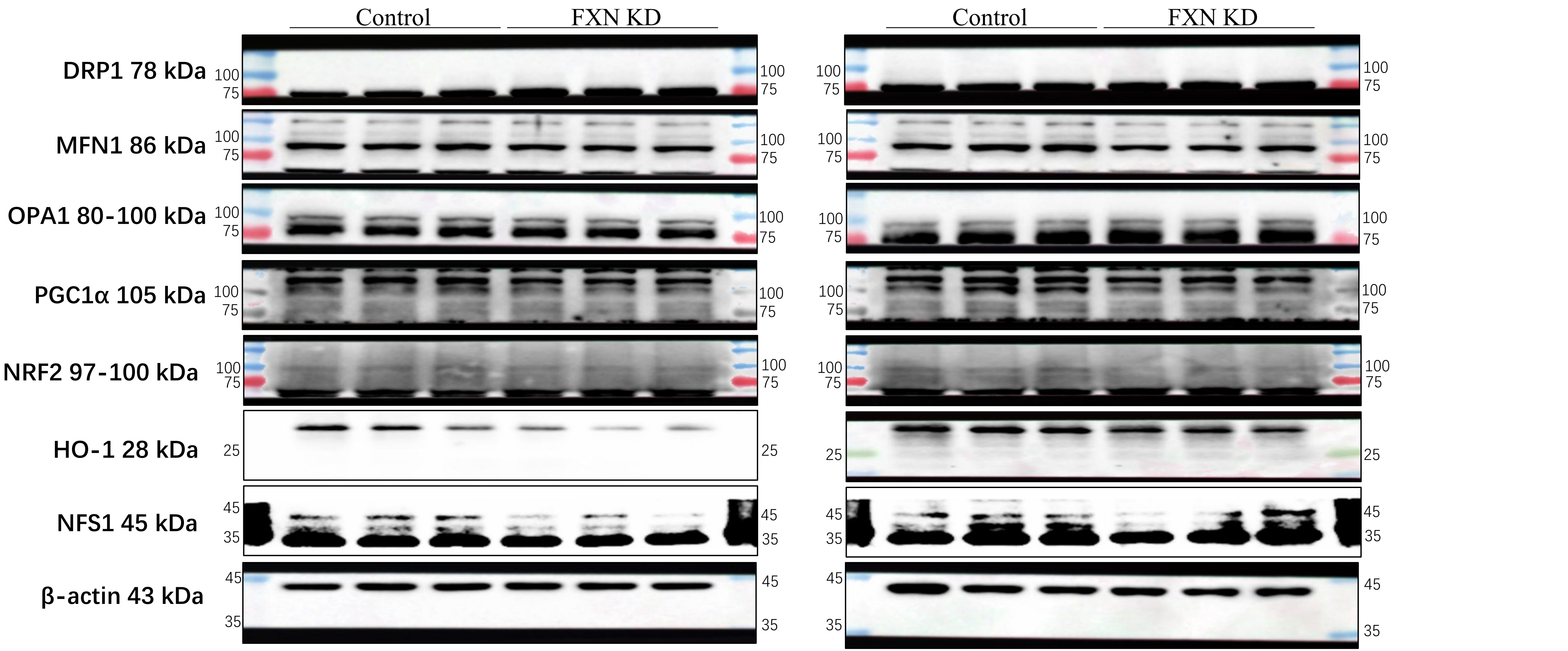
N2a cell lysate
Control
FXN KD
DRP1 78 kDa
MFN1 86 kDa
OPA1 80-100 kDa
PGC1α 105 kDa
NRF2 97-100 kDa
HO-1 28 kDa
NFS1 45 kDa
β-actin 43 kDa
100
75
25
45
35

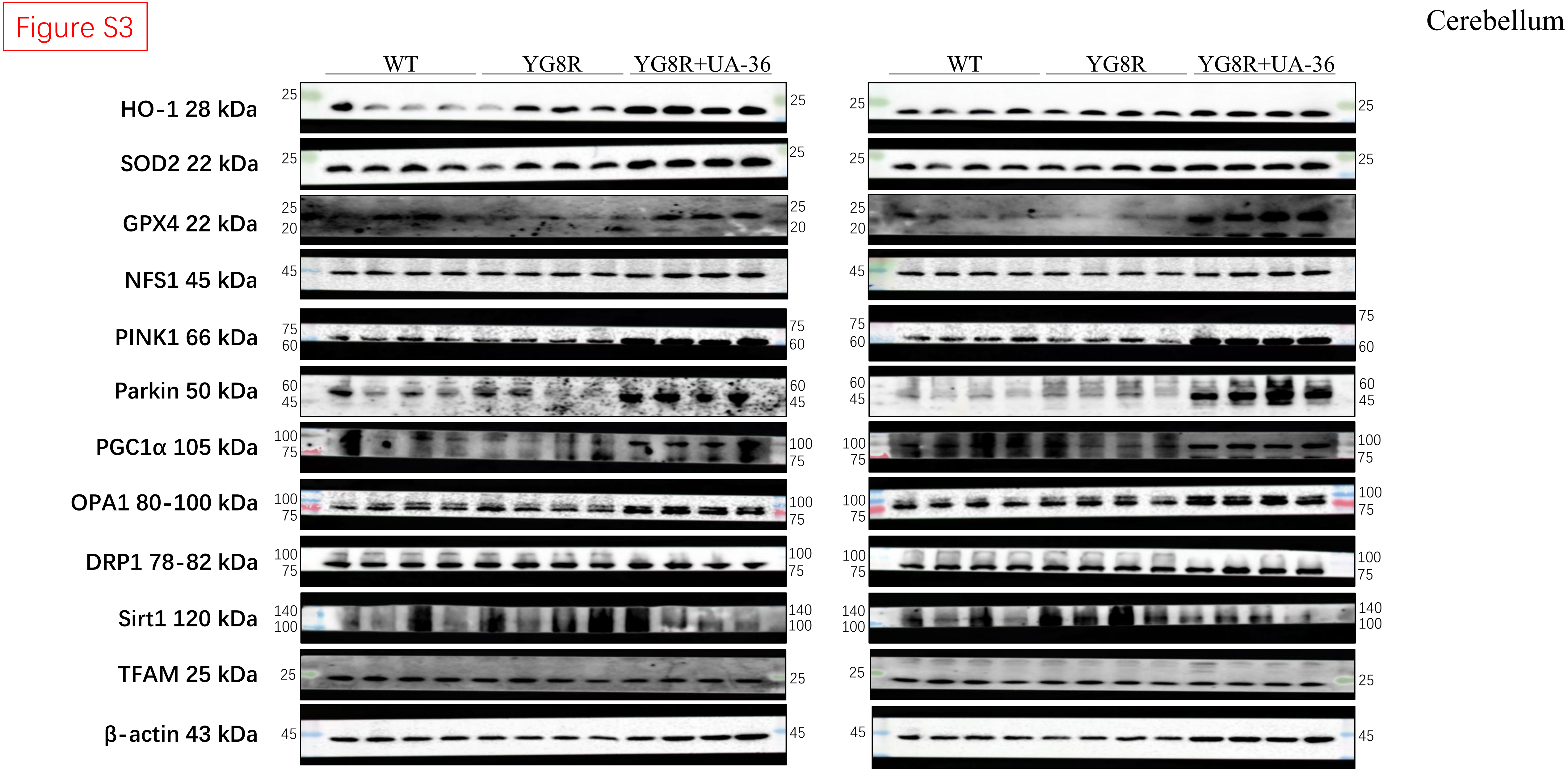
Figure S3
Cerebellum
WT
YG8R
YG8R+UA-36
HO-1 28 kDa
SOD2 22 kDa
GPX4 22 kDa
NFS1 45 kDa
PINK1 66 kDa
Parkin 50 kDa
PGC1α 105 kDa
OPA1 80-100 kDa
DRP1 78-82 kDa
Sirt1 120 kDa
TFAM 25 kDa
β-actin 43 kDa
